# Gender and Narrative Writing in Elementary Schools: A Qualitative Study Supporting Inclusive Education

**DOI:** 10.12688/f1000research.171604.1

**Published:** 2025-11-26

**Authors:** Sri Wulan Anggraeni, Dadang Sunendar, Isah Cahyani

**Affiliations:** 1 Faculty of Education Sciences, Universitas Pendidikan Indonesia Fakultas Ilmu Pendidikan, Bandung, West Java, 40154, Indonesia; 2 Faculty of Teacher Training and Education, Universitas Buana Perjuangan Karawang, Karawang Regency, West Java, 41361, Indonesia; 3 Faculty of Language and Literature Education, Universitas Pendidikan Indonesia Fakultas Pendidikan Bahasa dan Seni, Bandung, West Java, 40154, Indonesia; 4 Faculty of Language and Literature Education, Universitas Pendidikan Indonesia Fakultas Pendidikan Bahasa dan Seni, Bandung, West Java, 40154, Indonesia

**Keywords:** elementary education, gender and literacy, gender stereotypes, narrative writing, inclusive education

## Abstract

**Background:**

This study investigates gender differences in narrative writing among primary school students to promote inclusive education, as narrative writing not only demonstrates technical skills but also reflects embedded social and cultural gender norms that can influence the development of literacy and equity in classroom practice. By examining these differences, this study aims to highlight how gender stereotypes can marginalize certain narrative styles and inform pedagogical strategies to foster diverse voices in Indonesian primary schools.

**Methods:**

A qualitative case study approach was used, which involved the analysis of 33 handwritten narrative documents from fifth graders (12 boys, 21 girls) in Indonesian primary schools, along with semi-structured interviews conducted with 10 selected students (5 boys, 5 girls) to explore their writing process and perceptions of gender norms. Data were analyzed using thematic analysis, focusing on key dimensions such as content, organization, language use, and writing conventions; Ethical consent was obtained from the institutional review body of Universitas Buana Perjuangan (062/LPPM/UBP/2025), and informed consent was obtained in writing from parents/guardians, with verbal consent from child participants.

**Results:**

The results revealed that male students’ narratives were predominantly action-oriented and linear, emphasizing independence, physical activity, and limited emotional depth, aligned with traditional masculinity norms, while female students produced stories rich in emotional expression, interpersonal relationships, and detailed settings, reflecting a more reflective and relational style associated with feminine norms; Women also show higher proficiency in organizational and writing conventions, achieving better cohesion and technical accuracy, although both genders exhibit technical errors that sometimes obscure the expressive power of their writing, revealing how gender bias can underestimate diverse forms of narrative.

**Conclusions:**

These findings underscore how gender stereotypes shape literacy practices, which have the potential to perpetuate inequalities in education, and recommend the implementation of gender-sensitive assessment rubrics, cross-gender collaborative writing exercises, and teacher training on critical literacy to support an inclusive environment that validates authentic student voices and promotes equity, contributing to broader gender studies and inclusive education policies in Indonesia and Surrounding.

## Introduction

Narrative writing is one of the fundamental literacy skills taught since elementary school. However, narrative writing is not just a technical activity of writing; it serves as an arena for the formation of self-identity, social expression, and cultural representation that is strongly influenced by gender construction. Narratives allow students to organize ideas, develop characters, and construct storylines creatively, while reflecting on their personal and social experiences ( Beach & Caraballo, 2021; Montanero & Madeira, 2019). Thus, the narrative becomes a mirror of how children understand and interpret the world around them, which is inseparable from the social and cultural context that shapes it ( Davidson, Walton, Kansal, & Cohen, 2017; Healey, 2025).

Various studies have shown that there is a difference in patterns in narrative writing styles between male and female students. Female student narratives tend to emphasize interpersonal relationships, emotional depth, and personal reflection, while male student narratives emphasize more external actions, events, and dynamics ( Ahmadi-Azad, 2015; Coffman & Marques, 2021). These differences are often considered to be a natural manifestation of gender differences, even though a number of studies indicate that these patterns are the result of the socialization process of gender-biased literacy and normative expectations inherent in the educational environment ( Francis, Read, Melling, & Robson, 2003; Hewitt, 2021).

Although studies on gender-based communication and literacy have developed ( Lin, Dowell, Godfrey, Choi, & Brooks, 2019), research that critically examines how cultural biases and gender normative expectations affect early childhood narrative writing is still very limited. This gap is important to fill because it has the potential to reinforce gender stereotypes and widen the gap in literacy achievement between male and female students. Certain narrative styles associated with certain genders are often perceived as more valuable or more “correct,” thus creating inequities in the validation of literacy competencies in the classroom.

At the primary school level, where the foundations of literacy and social identity are beginning to take shape, a critical understanding of gender constructs in literacy is critical to prevent the reproduction of social inequality and support inclusive and equitable education. Literacy education that is insensitive to gender bias can reinforce stereotypes and limit children’s potential expression according to their gender identity. Therefore, dismantling gender-based literacy stereotypes is a strategic step to create a more democratic learning space and respect the diversity of styles of expression.

Based on the gaps identified in the literature, this study seeks to answer the research question: “How do gender norms and social values influence the narrative writing styles of male and female elementary school students, and what are the implications of these differences for inclusive literacy education?”

This study aims to fill this gap by in-depth analyzing the narrative styles of male and female students in elementary school. A qualitative approach based on text analysis is used to explore aspects of narrative structure, character depiction, language use, and organization of ideas and emotions in students’ narrative writing. In this way, the research seeks to reveal that differences in narrative styles are not just individual choices but rather a reflection of social values and gender biases that are absorbed into the education system.

The original contribution of this research lies in the courage to critically and systematically examine gender bias in children’s literacy expressions, something that is still rarely touched in the context of basic education. The findings are expected not only to enrich the study of gender literacy and education but also to provide an empirical basis for more inclusive and equitable curriculum and teaching practice reforms, thereby reducing access inequalities and validating literacy competencies based on gender.

Thus, this research is not only academically relevant but also has significant practical implications for the development of inclusive education in Indonesia and the global context. Dismantling gender-based literacy stereotypes early on is an important step to ensure that every child, without exception, has an equal opportunity to develop optimally in the realm of literacy and social identity.

## Literature review

Studies on the relationship between gender and narrative writing skills have evolved in recent decades. Various studies consistently show that there is a difference in writing styles between male and female students. Male students tend to emphasize aspects of external actions and events, while female students display more emotional depth, interpersonal relationships, and personal reflection ( Griva, Semoglou, & Panteli, 2009; Miftahur Ridha, T. Silvana Sinar, & Zein, 2024). Female students also generally outperform males in handwriting fluency, self-efficacy, spelling, text length, and overall quality, while males are more prone to technical errors ( Almusharraf & Alotaibi, 2021; Cordeiro, Castro, & Limpo, 2018). These patterns highlight that differences are shaped not only by cognitive factors but also by gender socialization in education.

Cross-cultural studies confirm that such differences emerge early, with masculinity linked to action and femininity to affection ( Grysman, 2018; Odağ, 2013). Importantly, they are not purely biological but reinforced by gender-biased literacy practices, including differential teacher feedback, which affects boys’ motivation and self-efficacy ( Abdullah, Idrus, Gao, & Muhammad, 2024; González-Díaz, Lampropoulou, Parr, & Johnson, 2025; Peterson & Kennedy, 2006).

In Indonesia, research on gendered literacy is still limited and tends to address general achievement rather than narrative writing. Yet early critical literacy interventions can help dismantle stereotypes ( Whitford, 2023) and unconscious-bias training for educators improves awareness and diversifies reading material ( Davenport, Heslop, Padwick, & Shimwell, 2024). National policies such as *Peraturan Menteri Pendidikan Nasional No. 84 Tahun 2008* concerning Guidelines for the Implementation of Gender Mainstreaming in the Education Sector and the National Medium-Term Development Plan (RPJMN) 2015–2025 emphasize the importance of gender equality, including in basic literacy ( Bappenas, 2019; Carolina, 2019).

Studies further note that academic assessments often privilege reflective, emotionally rich narratives closer to female styles while undervaluing concise, action-oriented male narratives ( Beard & Burrell, 2010; Budziszewska & Hansen, 2020). From a theoretical lens, the critical literacy framework calls for deconstructing such hidden norms to empower diverse voices ( George Lee, 2020; Goar, 2021; Janks, 2013). while hegemonic masculinity and feminine scripts explain the cultural shaping of boys’ action-oriented and girls’ affective writing styles ( Fernández & Sartori, 2012; Jewkes et al., 2015).

Thus, a research gap remains in Indonesia, especially in elementary school contexts, where few studies integrate qualitative narrative analysis with quantitative scoring to link technical writing skills and gender norms. This study addresses that gap and offers practical insights for more inclusive literacy education.

## Method

### Research design

This study uses a critical qualitative approach with a case study design ( Creswell & Poth, 2018; Yin, 2017), which aims not only to describe the differences in narrative styles between male and female students but also to unravel the social and cultural structures that make up these differences. The qualitative approach is grounded in interpretive paradigms that emphasize understanding lived experiences and social constructions ( Denzin & Lincoln, 2017). This study analyzes how elementary school grade V students structure their narratives, including in terms of character depiction, plot structure, language use, and the relationship of these aspects to gender-based literacy stereotypes. The study was conducted in an Indonesian public elementary school in an urban environment in Karawang, where cultural norms influenced by traditional gender roles, such as boys as active adventurers and girls as relational caregivers, are still strong. his is in line with the policy of the Ministry of Education and Culture (2008), which issued a regulation, *Peraturan Menteri Pendidikan Nasional No. 84 Tahun 2008,* and SDGs Goal 4 and Goal 5 which support quality education and gender equality ( Bappenas, 2019). In addition, the 2015-2025 National Medium-Term Development Plan (RPJMN) policy focuses on improving gender equality in education, aiming to create equal opportunities for all students ( Carolina, 2019). These factors contextualize how societal expectations can reinforce or challenge gender stereotypes in narratives, which in turn shapes student writing expressions with more inclusive approaches and storytelling that supports gender equality.

### Participants

This study used two primary data sources: observation of 33 written documents and interviews with 10 students. The documents analyzed were student narratives, focusing on character depiction, plot structure, language use, and how these aspects relate to gender-based literacy stereotypes. The selection of these 33 documents was carried out purposively, ensuring that they reflected a variety of narrative styles and allowed for in-depth thematic analysis. This approach aligns with recommendations for small, purposive samples in educational qualitative research ( Creswell & Poth, 2018; Patton, 2015).

In addition to the document analysis, 10 students were selected for interviews using purposive sampling. The criteria for selection were based on the presence of distinctive gendered writing patterns in their narratives, which made them relevant for further exploration in line with the study’s focus. This purposive approach allowed the researcher to target participants who could provide rich, contextual insights into both the narrative writing process and the influence of gender in writing. The sample size of 10 students was considered sufficient to achieve depth of analysis while remaining manageable for detailed qualitative inquiry.

The participants were all from a single grade V class at a public elementary school in urban Karawang, Indonesia. Informed consent was obtained in writing from parents or legal guardians of all participants prior to data collection, explaining the study’s purpose, procedures, potential risks, and the right to withdraw at any time. For the minor participants, verbal assent was secured directly from the children to ensure their understanding and willingness, as written assent may be challenging due to their age and developmental stage, which aligns with ethical guidelines from the Declaration of Helsinki and the American Psychological Association (2017). Participation was voluntary, with students free to opt out without penalty, and all involved did so willingly without assignment coercion. During the data collection, participants provided explanations as needed for task clarification but did not contribute to the validation of data post-collection due to the written nature of the task. To ensure confidentiality, all participants were anonymized using pseudonyms (e.g., Student M1 for male student 1) throughout the analysis and reporting, and no identifiable information, such as names or exact locations, was disclosed.

### Data collection

The main instrument in this study is the task of writing narratives. Students are asked to write a narrative story with the theme “Vacation Experience.” Instructions emphasize the drafting of a complete narrative structure: introduction, conflict, climax, and resolution. However, the main focus is not only on technical quality but also on how the written narrative reflects self-representation, social relations, and the influence of gender norms in their written expression. This study uses the following categories of narrative analysis (see Table 1 for details).

**Table 1.** Aspects of writing a narrative.

Aspects	Indicator	Scale *			
		1	2	3	4
Content (40%)	Flow				
	Characterization				
	Setting				
	Point of View				
Organization (30%)	Structure				
	Cohesion and Coherence				
Language (20%)	Sentence Structure and Phrase Formation				
	Diction				
Grammar (10%)	Spelling and Punctuation				

This table outlines the key categories used in the narrative analysis, including introduction, conflict, climax, and resolution, to evaluate students’ narrative writing in terms of structure and thematic elements.

* Description: 4 (Excellent), 3 (Good), 2 (Satisfactory), 1 (Poor).

**Figure 1.**
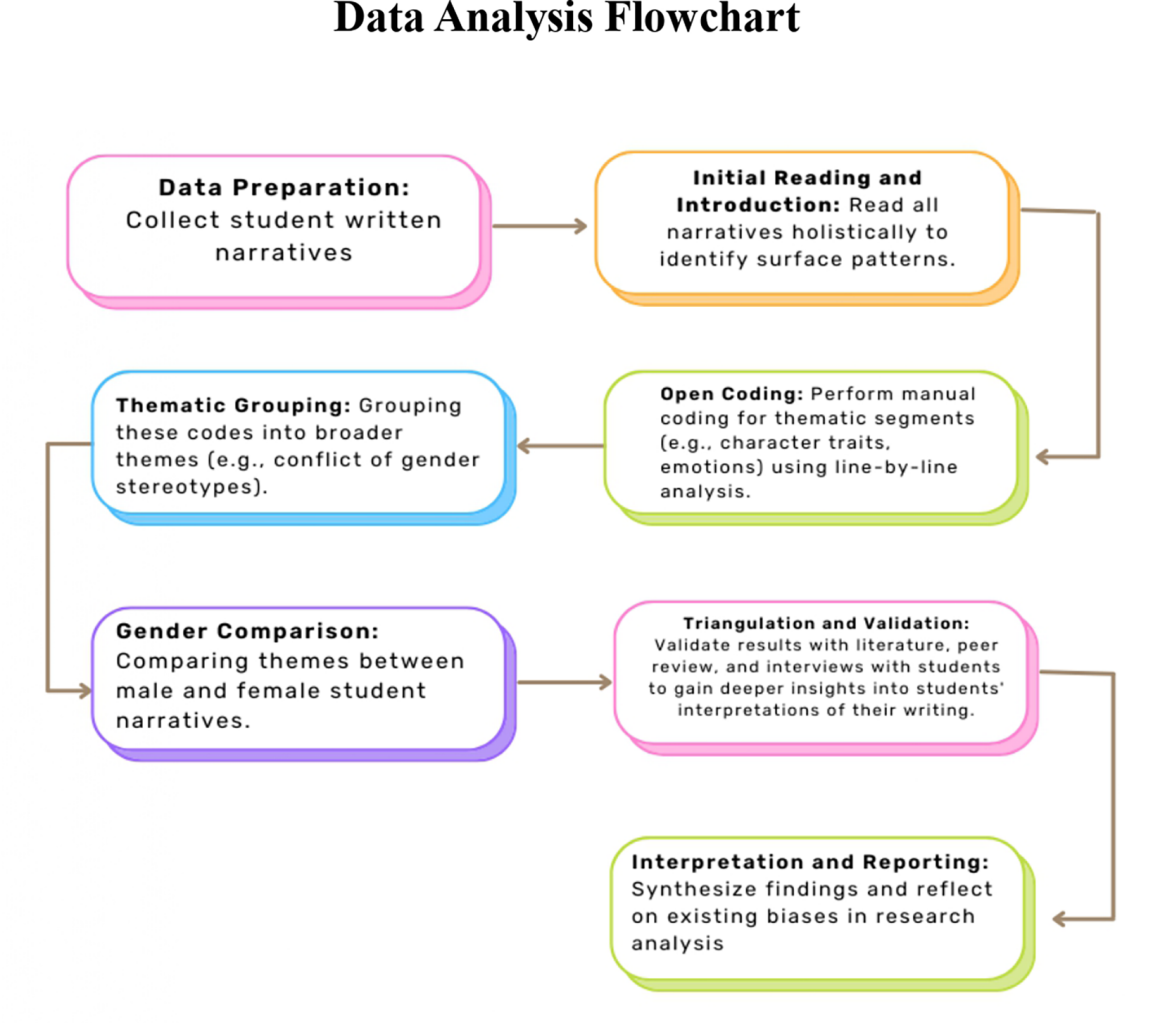
Data analysis flowchart. Figure 1 shows the analysis process following a systematic analysis using a critical thematic approach.

From the Table 1, the assessment of narrative writing ability using the formula:

$$\text{Value}=\frac{\text{Scores obtained in each aspect}\;}{\text{Maximum score of each aspect}}x\;\text{weight}\;\mathrm{on}\;\text{every aspect}$$

From the calculation of the formula above, the value in each aspect is cumulative so that the final value in writing a narrative can be obtained. This category was chosen not only as a technical tool but also as a means of reading the patterns of ideological expression implied in children’s writing.

The next data collection was conducted by researchers using interviews with 10 selected students to provide deeper insight into their narrative writing process. The data collection process involves recording and transcribing the results of the interview. Each interview is recorded using an audio recorder on a smartphone device. After the interview is over, the researcher transcribes the audio recordings to ensure that the data obtained can be analyzed carefully and accurately.

This transcription is then used to identify themes that emerge in students’ conversations about their experiences in writing narratives, as well as how they perceive gender norms in their writing. All transcripts are required to be anonymized to protect the privacy of the participants, using pseudonyms or identifier codes that do not reveal personal information such as the participant’s real name or location. The transcription process is carefully carried out to ensure that no information is missed or misinterpreted. After the transcription is complete, the researcher also verifies by re-reading the results of the interview and transcription to ensure the accuracy of the data collected.

### Data collection procedure

The data collection process was carried out for one week, from October 7 to October 12, 2024, with the task of writing a narrative given on October 3, 2024. Data collection was carried out by assigning narrative writing tasks to all students simultaneously in class for 60 minutes. Instruction is given orally and in writing to ensure students’ understanding of the assignment given. No changes were made to the planned procedures, as the original design had been adapted to the possible class conditions.

After the assignment is completed, the students’ writing is collected for further analysis. Handwriting is collected directly in the form of a physical document; there is no use of voice recorders or additional software during data collection. However, to obtain more in-depth data, interviews were conducted with 10 selected students to dig deeper into their experiences in writing narratives and their views on the influence of gender norms in their writing. Interviews are conducted in a structured manner with relevant questions to gain insight into the thinking process and students’ perspectives on research themes.

### Data analysis

The data analysis process follows systematic steps as listed below.

The data was analyzed using a critical thematic approach ( Braun & Clarke, 2006; Nowell, Norris, White, & Moules, 2017), It starts with open coding of all the results of narrative writing. The coding process is done manually without the use of software, through iterative reading in which segments of the text are highlighted and labeled to reflect the true meaning of the narrative.

To ensure accuracy, digital transcriptions of interviews are compared to the original handwritten data to verify the fidelity of the data, with no discrepancies recorded. Each narrative is analyzed to find dominant themes related to character depictions, emotions, social relationships, and the arrangement of conflicts and climaxes. This analysis not only stops at the linguistic and structural level, but is also geared towards identifying how gender norms are represented and reproduced in students’ writing.

Data from male and female students were then compared to explore patterns of expression dominance, thematic differences, and the marginalization potential of certain forms of expression. The analysis process is carried out in stages, starting from coding and grouping themes to comparisons between genders, to gain a comprehensive understanding.

Triangulation was conducted by comparing empirical findings with existing literature on children’s narratives and gender roles, such as research by Clarke, Marks, & Lykins (2016); Odağ (2013); Radina (2019) and others. Furthermore, validation was conducted through joint reading by several researchers of various text samples (involving two independent raters) to maintain objectivity of interpretation. Furthermore, student interviews were used as part of data triangulation to validate findings obtained from the analysis of written narratives. The interviews were conducted to delve deeper into the students’ perspectives on how they view gender norms reflected in their narrative writing. The interview results were compared with the findings of narrative writing to identify consistency or differences in their perspectives, which in turn provided deeper insights into the social factors that influenced their writing.

In addition, discussions and joint readings by several researchers were also carried out to ensure that the interpretation and analysis carried out were appropriate and acceptable. This increases the credibility of the analysis results ( Lincoln & Guba, 1985). Discussions between researchers were conducted to minimize subjective bias in data interpretation.

The data entry process is carried out by transcribing handwritten narrative writing manuals into password-protected digital files (stored on secure university servers with encrypted access and regular backups via Google Drive with two-factor authentication). Data security is managed by restricting access to only the research team, in accordance with Indonesia’s personal data protection regulations listed in Law ( *Law of the Republic of Indonesia Number 27 of 2022 on Personal Data Protection*, 2022) and deleting raw files after the analysis is completed. Anonymity is maintained by immediately encoding the data, ensuring no private identifiers remain in the dataset.

### Ethical consideration

The research and community service institute of Universitas Buana Perjuangan Karawang has approved this research with the contract number: 062/LPPM/UBP/2025. The researcher gave a letter of approval has also been given by the researcher to all respondents. Written consent to participate from the respondent was obtained in accordance with document 400.3.5/110/SD/2025. Respondents gave their consent without force from anyone. Subsequently, in order to protect the rights and privacy of the respondents, all forms of data acquired will remain confidential.

## Results

Students’ narrative writing is analyzed not only based on technical structure but also as a reflection of self-representation, social relationships, and gender norms that shape their mindset in expressing experiences. The narrative writing scores were obtained from 33 students whose assessments were based on the following aspects of narrative writing (see Table 2 for details).

**Table 2.** Narrative writing scores based on student narrative writing.

Aspects of narrative writing		Narrative writing of male student		x̄ Male	Narrative Writing of Female Students		x̄ Female
		Assessor 1	Assessor 2		Assessor 1	Assessor 2	
Content (40%)	x̄ Plot	2.6	2.2	2.4	3.0	3.0	3.0
	x̄ Characterization	2.0	2.0	2.0	2.0	2.0	2.0
	x̄ Setting	2.2	2.2	2.2	2.7	2.7	2.7
	x̄ Point of View	3.8	3.8	3.8	3.8	3.8	3.8
	**x̄ Content**			**26.5**	**x̄ Content**		**28.7**
Organization (30%)	x̄ Structure	2.0	2.8	2.4	3.0	3.0	3.0
	x̄ Cohesion and Coherence	2.0	2.4	2.2	2.6	3.0	2.8
	**x̄ Organization**			**17.5**	**x̄ Organization**		**21.8**
Language (20%)	x̄ Sentence Structure and Phrase Formation	2.3	2.7	2.5	2.5	2.5	2.5
	x̄ Diction	2.5	2.7	2.6	2.5	2.7	2.6
	**x̄ Language**			**12.7**	**x̄ Language**		**12.9**
Writing Conventions (10%)	x̄ Spelling and Punctuation	1.2	1.8	1.5	2.0	2.0	2.0
	**x̄ Writing Conventions**			**3.75**	**x̄ Writing Conventions**		**5.1**
	**x̄ Final Score**			**60.4**	**x̄ Final Score**		**68.5**

**Note**: The above data is taken from the observation of the narrative writing of 33 students. Full citation: Anggraeni, Sunendar, & Cahyani (2025d). *Results of observations of male and female students' narrative writing* [Dataset]. Figshare. https://doi.org/10.6084/m9.figshare.30281671.v1.

This table presents the average scores from 33 students (assessed by 2 assessors), comparing male and female students across aspects of narrative writing: content, organization, linguistic, and writing mechanics.

Based on the results of Table 2. The analysis of narrative writing assessed by 2 assessors, there is a significant difference between male and female students in several aspects of narrative writing. In the content aspect, which includes plot, characterization, setting, and point of view, female students obtained a higher average score, namely 28.7 compared to 26.5, indicating that female students tend to be more able to develop story content with a more elaborate plot and richer setting, although in the aspects of characterization and point of view, the scores are relatively the same. In the organizational aspect, which includes the structure of the essay as well as cohesion and coherence, the average score of female students was also superior, at 21.8 compared to 17.5 for male students, indicating that female students are better able to compose essays with a clear structure and more cohesive relationships between sentences. In the linguistic aspect, which includes the use of sentence structure and diction, the scores of the two groups of students were relatively balanced, with an average score of 12.9 for women and 12.7 for boys, indicating that the ability to use language in writing narratives was not too different between the two groups. Finally, in the aspect of writing, which includes the use of spelling and punctuation, female students again showed superiority with an average score of 5.1 compared to 3.75 for male students, indicating that female students are more careful in paying attention to the technical aspects of writing. Overall, the final score of narrative writing for female students was higher, which was 68.5 compared to 60.4 for male students.

### Story content

**Figure 2.**
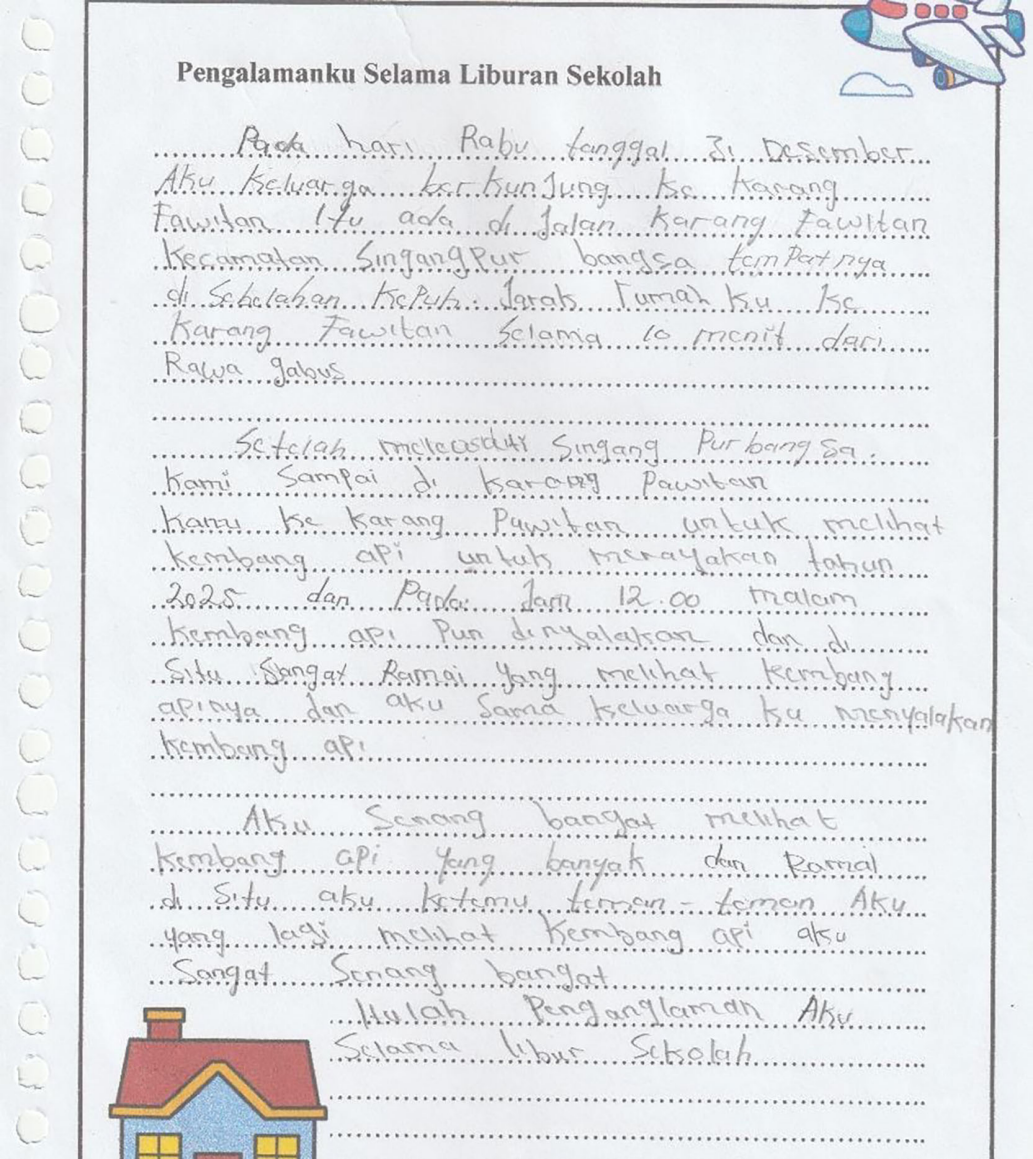
Male student narrative writing results (This visualization is based on the dataset available at https://doi.org/10.6084/m9.figshare.30353890.v1).

Male student writing tends to feature an action-oriented narrative, with a focus on physical activities such as playing ball, swimming, and everyday activities ( Anggraeni, Sunendar, & Cahyani, 2025a). Their stories show a linear, simple plot with a plot score of 2.4 and often without conflict or strong resolution. Characters in the story are only mentioned without character development or deep emotional expression, which is reflected in the characterization score of 2.0. The following is an example of the results of the narration writing of male students shown in Figure 2.

The writing of male students shows a tendency to construct stories based on repetitive and linear series of physical activities, as in the quote, “My sister and I were swimming. My sister and I played on slides. My sister and I bought sausages…” This plot does not show any climax, conflict, or emotional reflection. The narrative stops at reporting activities without depth of meaning. This reinforces the view that male narrative styles are formed to show ‘liveliness,’ not ‘feelings.’ The setting is used only as an action support, with no depiction of the atmosphere, which is reflected in the background score of 2.2. This is a common pattern of male narratives that do not place value on an emotional or atmospheric setting. In addition, the narrative of male students shows that the representation of the figure of ‘me’ is only portrayed as a perpetrator of the activity, without reflection of feelings or relational involvement. This reflects the form of narrative shaped by masculinity norms that emphasize independence, physical strength, and minimal emotional expression. One of the male students stated in an interview: *“I prefer to write about playing football or swimming because that’s what I do the most. Usually, I just tell what happened without thinking too much about the feelings or relationships between the characters. That’s not really important in my opinion.” *( Anggraeni, Sunendar, & Cahyani, 2025b).

**Figure 3.**
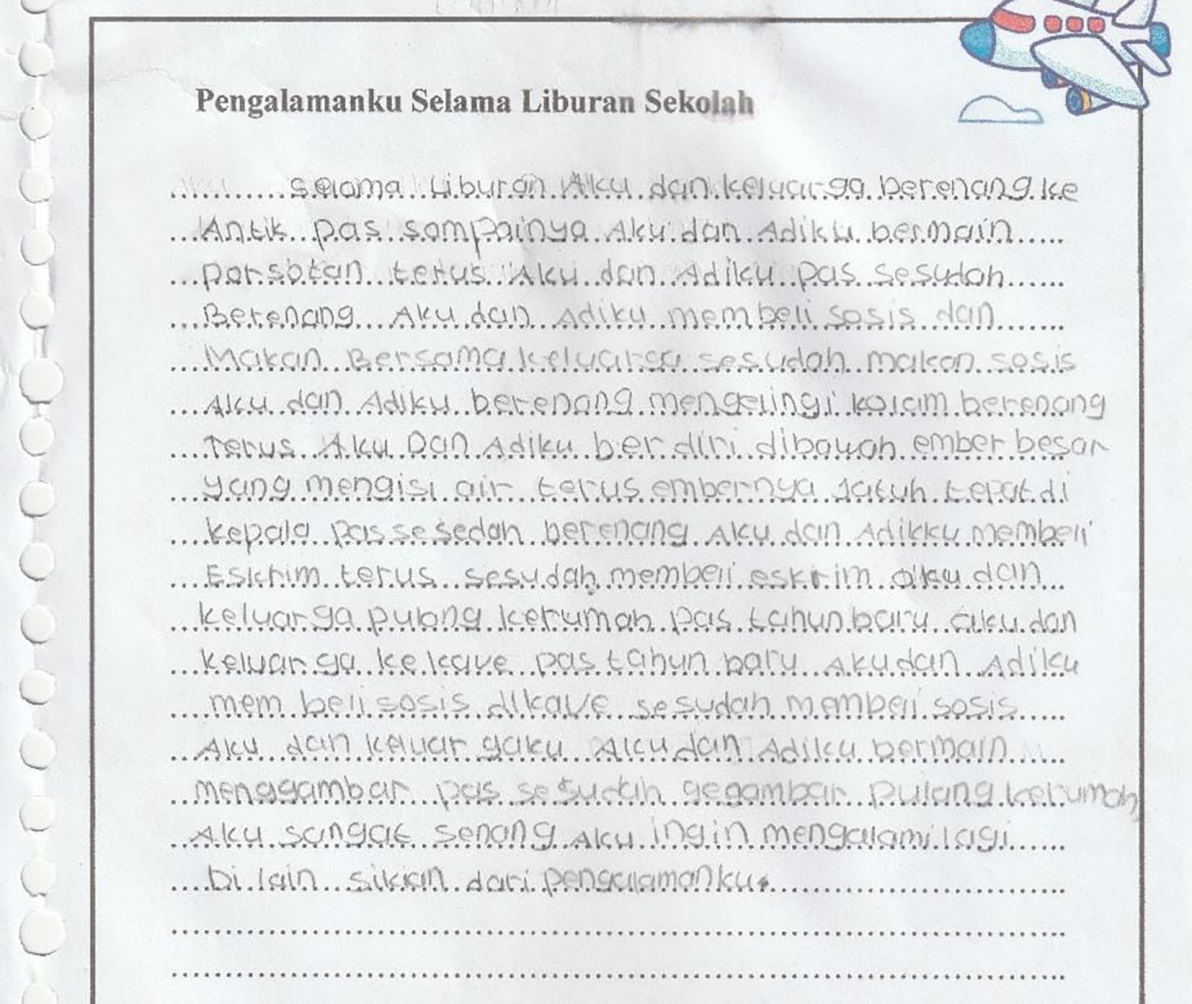
Results of narrative writing for female students (This visualization is based on the dataset available at https://doi.org/10.6084/m9.figshare.30353890.v1).

In contrast, female students’ narratives show a tendency toward emotional expression, depiction of atmosphere, and deeper interpersonal relationships. The main character not only performs activities but also establishes social relationships and portrays feelings in certain situations. This shows that self-expression in their writing is more influenced by affective values and feminine norms such as empathy and attachment ( Anggraeni et al., 2025a). As one female student stated: *“I love writing about my vacation experiences with my family because it makes me feel happy.”* ( Anggraeni et al., 2025b) *.* This is supported by a higher narrative content score of female students, which is 28.7 compared to male students, who are 26.5. The following is an example of the narrative writing of female students shown in Figure 3.

The narrative writing of female students in Figure 3 can build a plot with a time and emotion orientation. This is reflected in the flow score of 3.0 and the background score of 2.7. It exhibits a reflective and expressive narrative style, which is culturally more attached to the feminine role: organizing, paying attention to the atmosphere, and expressing feelings. As in the excerpt of the story piece “On Wednesday, December 31st… We went to Karang Pawitan to see the fireworks to welcome 2025… I’m so happy to see so many fireworks…”

This narrative shows the orientation of time, place, atmosphere, and personal feelings. This narrative style is more expressive and relational. The characterization of the narrative of women who have a characterization score of 2.0 emphasizes relationships and social connections, not just actions. This supports the assumption that women’s narratives often place them in a social context, rather than as independent individuals like male narratives. When viewed from the setting, female students are more involved in the emotional and sensory aspects of the reader, a narrative strategy that rarely appears in male student narratives. This suggests that female students are more sensitive to the emotional and aesthetic context of events ( Anggraeni et al., 2025a).

### Organization and cohesion

The writing of male students shows a fragmented narrative structure, with jumps between activities without strong logical transitions. Coherence between sections is often broken, reflecting event-oriented narrative processing rather than reflection or character development. This is reflected in the essay structure score of 2.4 and cohesion and coherence of only 2.2, which is a lower score than female students. This can be understood as part of a ‘masculine’ narrative construction, which prioritizes action over relationships. As in the phrases “Me and my sister swim…” “My sister and I bought sausages…” and “My sister and I play…” There is a lack of structural variation; it tends to be mechanical. Sentences do not develop semantically. This style is often considered ‘less attractive’ academically, but it may reflect a more “straight to the core” male communication pattern that is unfortunately often not valued in literacy curricula.

This finding is supported by interview data, where one male student stated:

*“I don’t really like writing complicated stories. I prefer a story that is fast and straight to the point. If something is interesting, I write it straight away without much thought about how the story should proceed.” *( Anggraeni et al., 2025b) *.*

Such a statement reinforces the idea that the fragmented structure is not merely a technical weakness but also a deliberate narrative choice aligned with a preference for brevity and directness.

Meanwhile, female students showed the ability to organize narratives in a more orderly manner, although some stories still ended abruptly. This is supported by a higher essay structure score of 3.0 and cohesion and coherence of 2.8. The cohesion of ideas and emotions is more evident in their narratives, showing an awareness of the need to unite social and mental experiences in a single story. In the narrative of the female, there are enough connectors of ideas (“after that,” “we got to…”) indicating that there is an effort to build a cohesive narrative even though it is not technically perfect. The emphasis on the continuity of events and emotions is a hallmark of narratives that are sensitive to social relations and feelings, in contrast to masculine narrative styles that are often discrete and change topics abruptly.

Interview evidence supports this interpretation. One female student explained:

*“When writing stories, I always try to make sure that all parts of the story are well connected. I think a good story should have a clear plot and describe how the characters in each section feel.”* ( Anggraeni et al., 2025b) *.*

This shows that female students have a conscious awareness of narrative flow and emotional coherence, which translates into higher organization and cohesion scores.

### Language and diction

Male students’ writing features short sentence structure, simple diction, and a lack of emotional variety. This is reflected in the sentence structure usage score of 2.5 and diction of 2.6, which shows a concise and action-oriented form of communication. As in the example of the male student’s narrative in Figure 2, there are many repetitions of words, for example, the words “swimming,” “playing,” and “me and my sister.” This shows that the sentence structure is very simple. Words are chosen based on actions, not reflections or expressions. This shows the role of gender stereotypes in language choices, where boys tend to avoid emotional expressions that are considered feminine.

This interpretation is supported by interview data. One male student expressed:

*“I usually write simple words. When I write my feelings, I am confused about how to write them. So I’m just telling you what I did.” *( Anggraeni et al., 2025b) *.*

This statement indicates that boys’ choice of simple, repetitive diction is not only a technical limitation but also a reflection of discomfort with emotional vocabulary, aligning with the masculine norm of emotional restraint.

In contrast, female students tend to use more expressive diction, emotional verbs, and sentence formulation that contains evaluations of situations. This is supported by slightly higher sentence structure and diction usage scores, 2.5 and 2.6, respectively, with a tendency to use expressive words such as “I’m really happy…” and “very crowded…” This diction is simple, but there is an attempt to explain the mood. Female student narrative writing uses more expressive and repetitive words, showing that this writing is directed to express emotions and experience involvement, not just record actions. This supports the finding that female narratives emphasize subjective experiences and emotional attachment, something that is less found in the writing of male students.

The interviews with female students corroborate this finding. One female student explained:

*“When I write stories, I like to write what I feel so that the reader knows I’m happy or sad. So I choose words that can show feelings.”* ( Anggraeni et al., 2025b) *.*

This response shows that female students have greater intentionality in choosing words that evoke feelings and atmosphere, reinforcing their higher scores in narrative expressiveness.

### Writing conventions

Both male and female students show technical errors, such as spelling and punctuation. This is reflected in the relatively low score of spelling and punctuation use, which is 1.5 for male students and 2.0 for female students. However, this error often masks the potential for greater narrative depth, especially when the narrative is read only from the grammatical aspect. It is important to note that the assessment of students’ work is not biased only on academic technical aspects so that it can provide space for richer and more diverse narrative expression.

### Gender norms and stereotypes in narrative writing

Based on the study of the results of narrative writing, it reveals how gender norms and stereotypes are implicitly represented and reproduced in student writing. The narrative of male students tends to assert traditional masculinity norms that emphasize independence, physical activeness, and emotional control. The ‘me’ figure in male narratives is more often portrayed as an actor without deep emotional involvement, which reflects social expectations for men to appear strong and not affectively expressive ( Anggraeni et al., 2025a).

This interpretation is reinforced by interview data. One male student stated:

*“I rarely write about feelings because I think it’s weird. It’s better for me to tell my story about what I do, like play ball or go swimming.” *( Anggraeni et al., 2025b) *.*

This quote illustrates how masculine identity construction leads boys to avoid affective expressions and focus on actions, aligning with hegemonic masculinity norms that value independence and physical activity.

In contrast, female student narratives feature feminine norms that emphasize social attachment, empathy, and emotional expression. Characters in women’s narratives not only play an active role but also show a connection with others and a rich reflection of feelings.

A female student shared during the interview:

*“I like to write stories that have a feeling of sadness or joy. If there are no feelings, the story is like a lack of life.” *( Anggraeni et al., 2025b) *.*

This statement confirms that girls see emotions as an integral part of storytelling, reflecting cultural expectations that validate empathy and relational sensitivity.

These findings suggest that even though students write based on personal experiences, they are still influenced by the social and cultural constructs that shape their understanding of gender roles. The interviews demonstrate that boys’ avoidance of emotional vocabulary and girls’ preference for emotive and relational narratives are not purely individual tendencies but are socially reinforced patterns.

### Gendered narrative styles

The comparison between the narratives of male and female students shows a different pattern of dominance of expression, where the male narrative style is more oriented towards external actions and events, while the female narrative style emphasizes interpersonal relationships and emotional depth. This pattern reflects not only individual preferences but also the potential marginalization of certain styles of expression in educational contexts. For example, more direct and simple male narratives are often undervalued in literacy assessments that prioritize the emotional depth and complexity of the narrative, which tend to be associated with female styles ( Anggraeni et al., 2025a).

Interview data provides additional insight into this phenomenon. One male student stated:

*“I like stories that end quickly, like telling me to play soccer, then go home. If I have to write long and have a lot of feelings, I get lazy.” *( Anggraeni et al., 2025b) *.*

This quote highlights that brevity and action-focus are conscious narrative choices rather than merely technical limitations, suggesting that boys value efficiency and directness in their storytelling.

Conversely, a female student shared:

*“I love that my story can make others feel what I feel. So I wrote it down so that the reader can imagine the atmosphere.”* ( Anggraeni et al., 2025b).

This illustrates that girls intentionally craft narratives to evoke empathy and emotional engagement from readers, which aligns with their higher scores in content and organization.

This pattern has implications for the reproduction of gender inequality in literacy education, where masculine expressions can be marginalized or considered less valuable when assessment criteria favor detailed, emotionally rich narratives. These findings are in line with critical literacy theory that emphasizes the importance of critiquing hidden norms and values in literacy practice, as well as gender studies that show how education can reproduce or challenge gender stereotypes.

## Discussion

The novelty of this research lies in its integrated approach to analyzing primary school students’ narrative writing in an Indonesian educational context, combining quantitative scoring from dual assessors with qualitative thematic analysis to uncover how gender norms and stereotypes are reproduced through self-representation, social relationships, and emotional expression. Unlike prior studies that primarily focus on Western adolescent or adult populations (e.g., Grysman, 2018; Odağ, 2013), this study pioneers an examination of young elementary students (aged 10-12) in a non-Western setting, highlighting the early onset of gendered narrative styles influenced by local cultural constructs like hegemonic masculinity and feminine empathy. It also proposes practical pedagogical interventions, such as gender-cross writing exercises and bias-reflective rubrics, to foster inclusive literacy education, addressing a gap in transformative applications of critical gender theory in primary EFL classrooms.

Male student narrative writing tends to focus on physical activities and daily activities, such as playing ball or swimming, with a simple plot and minimal climax. The characterization is more superficial, emphasizing the action rather than the feelings or relationships between the characters. This situation is in line with the concept of *hegemonic masculinity*, which is a dominant form of masculinity that emphasizes action, physical dominance, and emotional suppression as non-masculine traits ( Bartholomaeus, 2012; Jewkes et al., 2015). In the context of primary education, this norm leads boys to develop a linear and action-oriented narrative style while distancing them from the expression of affection and social reflection ( Duncanson, 2015). This kind of approach often ignores the emotional dimension that is essential for the development of complex narratives ( Bradford Wainwright, 2019). For example, in a family vacation story there is no clear resolution, indicating a focus on physical events without leaving room for emotional reflection. This reflects a masculine habitus that emphasizes physical independence and dominance of actions as key values in the formation of gender identity ( Elliott, Roberts, Ralph, Robards, & Savic, 2022; Sims-Gould et al., 2018). This kind of narrative often ignores the affective aspect due to social values that associate the expression of emotions with weakness, which is contrary to the dominant masculinity construct that begins to take shape since puberty ( Goldstein, 2003).

In addition, male students’ narrative writing often lacks a climax or resolution, confirming their tendency to write based on the flow of events rather than the flow of evolving conflict. This narrative style is driven by a higher engagement with action-type texts and linear structures without reflective depth ( Odağ, 2013). In addition, the socialization process that internalizes masculine norms also forms a narrative that is chronological and not emotionally problematic ( Radina, 2019). From the perspective of narrative theory, such texts tend to lose the elements of transition and dynamics, which are essential markers of a complete narrative ( Simon-Shoshan, 2013). Comparative studies show that men are more involved with action-oriented texts, while women with action-oriented texts are inward-experiential ( Odağ, 2013). This difference is also related to higher empathy and emotional intelligence scores in women ( Clarke, Marks, & Lykins, 2016), as well as social patterns that allow women to express affection ( Walton, Coyle, & Lyons, 2004).

On the other hand, the writings of female students show the depth of social relationships and moods. Their narratives highlight emotional interaction and thematic coherence ( Grysman, 2018; Schulkind, Schoppel, & Scheiderer, 2012). This situation reflects a “feminine script” that values empathy and social connection. This style is parallel to the concept of *l’écriture féminine*, which is a feminine writing style that focuses on affective experiences, emotional expression, and human relationships ( Fernández & Sartori, 2012). Psychologically, this tendency is also supported by the finding that women generally have higher levels of cognitive and emotional empathy than men ( Demir, Atli, & Kis, 2016; Karniol & Čehajić-Clancy, 2020). This contributes to a more reflective and relational narrative structure in female students’ writing, reinforcing the meaning of events through feelings, not just action sequences.

Another difference lies in the cohesion and organization of the story. Male student writing tends to jump between actions without obvious logical connectors. This style is often considered structurally “disconnected,” but it can be read as a form of cohesion typical of masculine groups that prioritize action over reflection or the connectedness of ideas. Research shows that in social contexts and collaborative learning, writing styles can develop as a product of group interaction and internal social pressures ( Schipke, 2005). In addition, educators often view men’s writing as more concise but less coherent than women’s, a perception that confirms a bias against action-based narrative styles ( Francis et al., 2003). Weaknesses in the use of cohesion devices were also found in general in EFL students, which reinforced the discourse that this “jump-hop” style is the result of an interaction between gender norms and technical skills ( Albelihi, 2022). In contrast, female narratives use temporal connectors (“after that,” “we got to…”) so that they are smoother, although sometimes at the expense of fluidity for order ( Schulkind et al., 2012). Their “narrative of emotional closeness” strategy is in line with Odağ’s (2013) findings about reader engagement through affection.

Linguistic errors appear in both groups, but they take different forms. In male students, diction selection tends to be simple and repetitive. This style is not just a linguistic limitation but rather a reflection of masculine norms that avoid explicit emotional expression. Patriarchal culture forms the narrative that affection is a weakness, so men learn to avoid affective verbs ( Allan, 2016; McQueen, 2017). In this context, repetition is not just a technical error but a form of emotional sublimation that should not be said directly. In contrast, female students’ writing showed a tendency to use emotionally oriented verbs such as “pleased,” “moved,” or “afraid,” accompanied by details that brought their narrative experience to life. This style exhibits a higher level of expressiveness than male students, as demonstrated by the use of direct and dramatic evaluations in their narratives ( Bohanek, Fivush, & Walker, 2005; Nureldeen, Alsabatin, Eskander, & Nasr, 2023).

Spelling and punctuation errors not only disrupt the clarity of the writing but also directly affect the way readers assess competence and trust in the author. ( Johnson, Wilson, & Roscoe, 2017) show that texts with technical errors are considered to be of lower quality, and the authors are considered less competent. Additionally, Witchel et al. (2020, 2022) found that errors such as misspellings and improper capitalization lower the perception of trust in information, even in critical contexts such as health forums. However, this mechanical judgment had a greater impact on male texts whose sentences were short and interrupted so that there appeared to be “more” errors even though the length of the text was shorter.

The difference in narrative style between male and female students is not a reflection of individual abilities or sensitivities alone, but rather the result of social constructions shaped by family, school, and assessment systems. Family and popular culture instill scripts that “men act, women feel,” while schools reproduce this script through assessment rubrics and sample texts that tend to affirm masculine expression as the default standard. A national grading system that focuses on technical criteria without considering a variety of expressions actually strengthens the hierarchy of writing styles. As a result, male students are often rewarded for their concise though superficial writing, while female students are praised for being detailed but are still criticized for being long-winded. This is a paradox of literacy stereotypes that not only corners one gender but also limits the authentic expression of both.

To respond to this, a more equitable and transformative pedagogical approach is needed. Teachers can start by designing a double rubric that separates the technical and expressive aspects so that emotional expression and reflective narratives are not sidelined by mechanical errors. Gender cross-writing exercises are also important to encourage the exploration of new styles, such as male students writing affective narratives and female students writing action reports. In addition, providing text models from different genres and gender perspectives can open students’ horizons to the diversity of literacy expressions. Teachers also need to be reflective of their own biases when giving feedback, as comments like “short but clear” or “neat and sweet” can reinforce narrow gender norms. With these steps, the classroom can become a place for deconstructing stereotypes and strengthening inclusive literacy.

## Conclusion

This study addresses its aims and research questions by qualitatively and quantitatively analyzing gender narrative styles among 33 fifth-grade Indonesian elementary school students (aged 10–12), revealing how gender norms shape self-representation, social relationships, and emotional expression in literacy practices, with implications for inclusive education. Findings indicate narratives are influenced by technical skills and early social constructions: per hegemonic masculinity ( Bartholomaeus, 2012; Jewkes et al., 2015), which prioritizes action and emotional control, boys’ writing is linear and activity-focused, scoring lower in content (26.5) and organization (17.5); conversely, per feminine script and l’écriture féminine ( Fernández & Sartori, 2012), which emphasizes empathy and affective relationships, girls’ narratives highlight emotion and connection, scoring higher in content (28.7), organization (21.8), and conventions (5.1). These patterns, from the analysis of holiday stories, reflect family, cultural, and school socialization that may unintentionally marginalize masculine styles when assessments favor emotionally rich and highly structured narratives.

In line with critical literacy theory, which critiques hidden norms to enable transformative practices, these inequities limit authentic expression and widen gaps. Educators should implement dual rubrics, cross-gender exercises, and reflective feedback on bias to validate diverse voices. From this Indonesian context, these findings can also inform other educational institutions particularly those in multicultural or gender-diverse environments seeking to promote equity through policies aligned with the National Medium-Term Development Plan (2015–2025) and SDGs 4–5. Eliminating bias early on empowers classrooms for optimal, stereotype-free development.

### Limitations and further research

This study has several limitations that should be acknowledged. First, the research was conducted in a single public elementary school in an urban setting, which may limit the generalizability of findings to rural schools or different socio-economic contexts. Second, the qualitative data were based on written narratives and interviews from a relatively small sample (33 students, 10 interviews), which, while sufficient for in-depth thematic analysis, may not capture the full diversity of narrative styles across Indonesia.

Future research should expand to multiple schools with diverse cultural and socio-economic backgrounds, employ larger and more balanced samples, and consider longitudinal designs to observe how gendered narrative styles evolve over time. Mixed-method approaches integrating quantitative linguistic analysis tools (e.g., corpus linguistics software) could provide additional objectivity. It would also be valuable to explore teacher perceptions and grading rubrics to determine how assessment practices directly shape and potentially reinforce these gendered narrative patterns. Finally, cross-cultural studies comparing Indonesian data with other countries in Southeast Asia could offer insights into whether these patterns are culturally specific or universal.

## Data Availability

- Figshare: ‘Analysis of Thematic Coding Results Based on Interviews’ Doi: https://doi.org/10.6084/m9. figshare.30283300.v1 ( Anggraeni et al., 2025a). which contains anonymized thematic coding and interview data necessary for replicating the qualitative analysis. - Figshare: ‘Interview Results of Gender and Narrative Writing’ Doi: https://doi.org/10.6084/m9. figshare.30281446.v2 ( Anggraeni et al., 2025b), including transcribed and anonymized interview excerpts for themes related to gender norms. - Figshare: ‘Results of Observations of Male and Female Students’ Narrative Writing:’ Doi: https://doi.org/10.6084/m9.figshare.30281587.v1 ( Anggraeni et al., 2025d), which provides observation data. - Figshare: ‘Narrative Writing by Male and Female Students’ Doi: https://doi.org/10.6084/m9.figshare.30353890.v1( Anggraeni et al., 2025c), which includes samples and figures for analysis, such as narrative points and themes.
