# Gender and Narrative Writing in Elementary Schools: A Qualitative Study Supporting Inclusive Education

**DOI:** 10.12688/f1000research.171604.2

**Published:** 2026-05-02

**Authors:** Sri Wulan Anggraeni, Dadang Sunendar, Isah Cahyani

**Affiliations:** 1 Faculty of Education Sciences, Universitas Pendidikan Indonesia Fakultas Ilmu Pendidikan, Bandung, West Java, 40154, Indonesia; 2 Faculty of Teacher Training and Education, Universitas Buana Perjuangan Karawang, Karawang Regency, West Java, 41361, Indonesia; 3 Faculty of Language and Literature Education, Universitas Pendidikan Indonesia Fakultas Pendidikan Bahasa dan Seni, Bandung, West Java, 40154, Indonesia; 4 Faculty of Language and Literature Education, Universitas Pendidikan Indonesia Fakultas Pendidikan Bahasa dan Seni, Bandung, West Java, 40154, Indonesia

**Keywords:** elementary education, gender and literacy, gender stereotypes, narrative writing, inclusive education

## Abstract

**Background:**

This study investigates gender differences in narrative writing among primary school students to promote inclusive education, as narrative writing reflects embedded gender norms that influence literacy development and classroom equity. By examining these differences, this study aims to highlight how gender stereotypes can marginalize certain narrative styles and inform pedagogical strategies for Indonesian schools.

**Methods:**

This qualitative case study with quantitative analysis examined 33 handwritten narratives from fifth-grade students (12 boys, 21 girls) and interviews with 10 students. SES data were collected through a parent questionnaire covering education, occupation, and home literacy environment. Two blind raters evaluated the narratives, with moderate inter-rater reliability (Cohen’s κ = 0.434). Thematic analysis was supplemented with t-tests and Pearson correlations to examine gender differences and the relationship between SES and narrative quality.

**Results:**

The results showed that female students excelled notably in organization (mean score 18,4 vs. 15,1 for males), grammar (9,95 vs. 8,15), and writing conventions (2,7 vs. 2,2), indicating greater coherence, grammatical accuracy, and technical precision in their narratives. In contrast, male students scored slightly higher on content (26 vs. 25,3), driven by a marginally higher focus on characterization, though females led in plot complexity and setting detail. Language scores were comparable between genders. The overall final narrative score favored females (68,95 vs. 63,95). Correlation analysis revealed significant relationships between maternal education, home literacy environment, and narrative quality.

**Conclusions:**

Gender stereotypes shape literacy practices and may perpetuate educational inequalities. The study recommends gender-sensitive rubrics, cross-gender writing exercises, and teacher training to support inclusive education that validates diverse student voices.

## Introduction

Narrative writing is one of the fundamental literacy skills taught since elementary school. However, narrative writing is not just a technical activity of writing; it serves as an arena for the formation of self-identity, social expression, and cultural representation that is strongly influenced by gender construction. Narratives allow students to organize ideas, develop characters, and construct storylines creatively, while reflecting on their personal and social experiences ( Beach & Caraballo, 2021; Montanero & Madeira, 2019). Thus, the narrative becomes a mirror of how children understand and interpret the world around them, which is inseparable from the social and cultural context that shapes it ( Davidson, Walton, Kansal, & Cohen, 2017; Healey, 2025).

Various studies have shown that there is a difference in patterns in narrative writing styles between male and female students. Female student narratives tend to emphasize interpersonal relationships, emotional depth, and personal reflection, while male student narratives emphasize more external actions, events, and dynamics ( Ahmadi-Azad, 2015; Coffman & Marques, 2021). These differences are often considered to be a natural manifestation of gender differences, even though a number of studies indicate that these patterns are the result of the socialization process of gender-biased literacy and normative expectations inherent in the educational environment ( Francis, Read, Melling, & Robson, 2003; Hewitt, 2021).

Although studies on gender-based communication and literacy have developed ( Lin, Dowell, Godfrey, Choi, & Brooks, 2019), research that critically examines how cultural biases and gender normative expectations affect early childhood narrative writing is still very limited. This gap is important to fill because it has the potential to reinforce gender stereotypes and widen the gap in literacy achievement between male and female students. Certain narrative styles associated with certain genders are often perceived as more valuable or more “correct,” thus creating inequities in the validation of literacy competencies in the classroom.

At the primary school level, where the foundations of literacy and social identity are beginning to take shape, a critical understanding of gender constructs in literacy is critical to prevent the reproduction of social inequality and support inclusive and equitable education. Literacy education that is insensitive to gender bias can reinforce stereotypes and limit children’s potential expression according to their gender identity. Therefore, dismantling gender-based literacy stereotypes is a strategic step to create a more democratic learning space and respect the diversity of styles of expression.

Based on the gaps identified in the literature, this study aims to answer the main research question: “How do gender norms and social values influence male and female students’ narrative writing styles, and what are the implications of these differences for inclusive literacy education?” To systematically address this question, this study addresses several sub-questions, namely: how do male and female students’ narrative content differ, including character portrayal, plot development, and thematic expression; differences in the organization and cohesion of their narratives; differences in the use of language and diction in narrative writing; the nature of errors in technical writing conventions (e.g., spelling, punctuation) in male and female students and their relationship to the overall quality of their narratives; and how these gender-influenced narrative differences can inform pedagogical strategies to promote inclusive literacy education.

This study aims to fill this gap by in-depth analyzing the narrative styles of male and female students in elementary school. A qualitative approach based on text analysis is used to explore aspects of narrative structure, character depiction, language use, and organization of ideas and emotions in students’ narrative writing. In this way, the research seeks to reveal that differences in narrative styles are not just individual choices but rather a reflection of social values and gender biases that are absorbed into the education system.

The original contribution of this research lies in the courage to critically and systematically examine gender bias in children’s literacy expressions, something that is still rarely touched in the context of basic education. The findings are expected not only to enrich the study of gender literacy and education but also to provide an empirical basis for more inclusive and equitable curriculum and teaching practice reforms, thereby reducing access inequalities and validating literacy competencies based on gender.

Thus, this research is not only academically relevant but also has significant practical implications for the development of inclusive education in Indonesia and the global context. Dismantling gender-based literacy stereotypes early on is an important step to ensure that every child, without exception, has an equal opportunity to develop optimally in the realm of literacy and social identity.

## Literature review

Studies on the relationship between gender and narrative writing skills have evolved in recent decades. Various studies consistently show that there is a difference in writing styles between male and female students. Male students tend to emphasize aspects of external actions and events, while female students display more emotional depth, interpersonal relationships, and personal reflection ( Griva, Semoglou, & Panteli, 2009; Miftahur Ridha, T. Silvana Sinar, & Zein, 2024). Female students also generally outperform males in handwriting fluency, self-efficacy, spelling, text length, and overall quality, while males are more prone to technical errors ( Almusharraf & Alotaibi, 2021; Cordeiro, Castro, & Limpo, 2018). These patterns highlight that differences are shaped not only by cognitive factors but also by gender socialization in education.

Cross-cultural studies confirm that such differences emerge early, with masculinity linked to action and femininity to affection ( Grysman, 2018; Odağ, 2013). Importantly, they are not purely biological but reinforced by gender-biased literacy practices, including differential teacher feedback, which affects boys’ motivation and self-efficacy ( Abdullah, Idrus, Gao, & Muhammad, 2024; González-Díaz, Lampropoulou, Parr, & Johnson, 2025; Peterson & Kennedy, 2006).

In Indonesia, research on gendered literacy is still limited and tends to address general achievement rather than narrative writing. Yet early critical literacy interventions can help dismantle stereotypes ( Whitford, 2023) and unconscious-bias training for educators improves awareness and diversifies reading material ( Davenport, Heslop, Padwick, & Shimwell, 2024). National policies such as *Peraturan Menteri Pendidikan Nasional No. 84 Tahun 2008* concerning Guidelines for the Implementation of Gender Mainstreaming in the Education Sector and the National Medium-Term Development Plan (RPJMN) 2015–2025 emphasize the importance of gender equality, including in basic literacy ( Bappenas, 2019; Carolina, 2019).

Studies further note that academic assessments often privilege reflective, emotionally rich narratives closer to female styles while undervaluing concise, action-oriented male narratives ( Beard & Burrell, 2010; Budziszewska & Hansen, 2020). From a theoretical lens, the critical literacy framework calls for deconstructing such hidden norms to empower diverse voices ( George Lee, 2020; Goar, 2021; Janks, 2013). while hegemonic masculinity and feminine scripts explain the cultural shaping of boys’ action-oriented and girls’ affective writing styles ( Fernández & Sartori, 2012; Jewkes et al., 2015).

Thus, a research gap remains in Indonesia, especially in elementary school contexts, where few studies integrate qualitative narrative analysis with quantitative scoring to link technical writing skills and gender norms. This study addresses that gap and offers practical insights for more inclusive literacy education.

## Method

### Research design

The study uses a critical qualitative approach combined with a case study design, which is well-suited to exploring complex social phenomena embedded in specific contexts ( Creswell & Poth, 2018; Yin, 2017). The case study method allows for an in-depth and holistic examination of the narrative writing practices of grade V students in their natural educational and cultural environments, allowing for rich and contextual understanding beyond quantitative measures.

The critical qualitative approach is based on an interpretive paradigm ( Denzin & Lincoln, 2017). That emphasizes the understanding of participants’ life experiences and the social constructs that influence those experiences. More importantly, this approach integrates a critical lens to interrogate and challenge power relations, social inequality, and hegemonic norms, such as gender constructions, that underlie literacy practices. This means that this study not only illustrates differences in narrative styles but aims to uncover the sociocultural structures and gender-based power dynamics that shape these differences and influence educational equity. The study was conducted in a public elementary school in Indonesia in an urban environment in Karawang, where cultural norms influenced by traditional gender roles, such as boys as active adventurers and girls as relational caregivers, are still strong. This is in line with the policy of the Ministry of Education and Culture (2008), which issued regulations, Regulation of the Minister of National Education No. 84 of 2008, and Sustainable Development Goals (SDGs) Goal 4 and Goal 5 that support quality education and gender equality ( Bappenas, 2019). In addition, the National Medium-Term Development Plan (RPJMN) 2015–2025 policy focuses on improving gender equality in education, aiming to create equal opportunities for all students ( Carolina, 2019). These factors contextualize how societal expectations can reinforce or challenge gender stereotypes in narratives, which in turn shapes student writing expressions with more inclusive and storytelling approaches that support gender equality.

### Participants

This study used two primary data sources: 33 student narrative documents and interviews with 10 students. The 33 narrative documents were purposively selected to represent a diverse range of narrative writing styles, enabling an in-depth thematic analysis aligned with recommendations for small, purposive samples in qualitative educational research ( Creswell & Poth, 2018; Patton, 2015).

From these 33 students, 10 were selected for semi-structured interviews using purposive sampling based on the presence of distinctive gendered writing patterns identified during document analysis. This purposive selection aimed to target participants who could provide rich, contextual insights about both the writing process and the influence of gender norms on their narratives. The choice of 10 interviewees was guided by the principle of data saturation, where no new themes emerged from additional interviews, ensuring a balance between depth of understanding and feasibility for detailed analysis. The representativeness of this subsample with regard to gender distribution (5 boys, 5 girls) also supports the validity of gender-focused comparisons.

To minimize the influence of *confounding variables* in gender-based interpretations, data on socio-economic status (SES) and home literacy environment were collected through a questionnaire of parents. The questionnaire instruments included indicators of parental education, parental work, number of books at home, frequency of reading activities, parental involvement in accompanying reading, access to the internet/devices, and participation in tutoring. This data allows contextualization of writing differences and helps control potential biases derived from socio-economic and environmental factors, which are known to affect children’s literacy development.

Based on the results of the questionnaire, the following characteristics of the participants were obtained:

Based on Table 1, the participants in this study were dominated by students from lower middle socio-economic backgrounds, with 63.6% in the Lower SES category and 27.3% in the Lower Secondary SES, while only 9.1% were included in the Upper Secondary SES and there were no students from the Upper SES category. Parents’ education mostly only reaches the elementary to high school level (94%), with father’s work dominated as farm laborers or factory workers (66.6%) and mother’s work as housewives (57.6%). The home literacy environment is also still limited, characterized by the ownership of books under 10 titles in 78.8% of students, low reading frequency (66.7% read less than 3 times per week), and lack of parental assistance in reading activities (75.8% rarely or never accompanied). This demographic condition provides an important context in understanding the results of students’ narrative writing, while at the same time emphasizing that any differences found between genders need to be analyzed by considering the limitations of this relatively homogeneous socio-economic background.

**Table 1.** Characteristics of respondents by gender.

Characteristics	Category	Male (n = 12)	Female (n = 21)	Total (N = 33)
Age	10 years	4 (33,3%)	7 (33,3%)	11 (33,3%)
	11 years	5 (41,7%)	9 (42,9%)	14 (42,4%)
	12 years	3 (25,0%)	5 (23,8%)	8 (24,3%)
SES	Low	8 (66,7%)	13 (61,9%)	21 (63,6%)
	Lower middle	4 (33,3%)	5 (23,8%)	9 (27,3%)
	Upper middle	0 (0%)	3 (14,3%)	3 (9,1%)
Father’s Education	Elementary School	4 (33,3%)	6 (28,6%)	10 (30,3%)
	Junior High School	3 (25,0%)	5 (23,8%)	8 (24,2%)
	Senior High School	4 (33,3%)	8 (38,1%)	12 (36,4%)
	Diploma	0 (0%)	1 (4,8%)	1 (3,0%)
	Bachelor	1 (8,4%)	1 (4,7%)	2 (6,1%)
Mother’s Education	Elementary School	5 (41,7%)	7 (33,3%)	12 (36,4%)
	Junior High School	3 (25,0%)	6 (28,6%)	9 (27,3%)
	Senior High School	3 (25,0%)	6 (28,6%)	9 (27,3%)
	Diploma	0 (0%)	1 (4,7%)	1 (3,0%)
	Bachelor	1 (8,3%)	1 (4,8%)	2 (6,0%)
Father’s Work	Farm workers	5 (41,7%)	9 (42,9%)	14 (42,4%)
	Factory/building workers	3 (25,0%)	5 (23,8%)	8 (24,2%)
	Small entrepreneurs	2 (16,7%)	4 (19,0%)	6 (18,2%)
	Private employees	2 (16,6%)	3 (14,3%)	5 (15,2%)
Mother’s Work	Farm workers	7 (58,3%)	12 (57,1%)	19 (57,6%)
	Factory/building workers	2 (16,7%)	4 (19,0%)	6 (18,2%)
	Small entrepreneurs	1 (8,3%)	1 (4,8%)	2 (6,1%)
	Private employees	1 (8,3%)	2 (9,5%)	3 (9,1%)
	Farm workers	1 (8,4%)	2 (9,6%)	3 (9,0%)
Number of Books at Home	< 5 books	6 (50,0%)	9 (42,9%)	15 (45,5%)
	5–10 books	4 (33,3%)	7 (33,3%)	11 (33,3%)
	11–20 books	2 (16,7%)	3 (14,3%)	5 (15,1%)
	21–50 books	0 (0%)	2 (9,5%)	2 (6,1%)
Reading Frequency	Almost never	5 (41,7%)	6 (28,6%)	11 (33,3%)
	1–2 times/week	4 (33,3%)	7 (33,3%)	11 (33,3%)
	3–4 times/week	2 (16,7%)	5 (23,8%)	7 (21,2%)
	Almost every day	1 (8,3%)	3 (14,3%)	4 (12,2%)
Parent Assistance	Never	6 (50,0%)	7 (33,3%)	13 (39,4%)
	Sometimes (1–2x/month)	4 (33,3%)	8 (38,1%)	12 (36,4%)
	Frequent (1–2x/week)	2 (16,7%)	4 (19,0%)	6 (18,2%)
	Every day	0 (0%)	2 (9,6%)	2 (6,0%)
Internet Access	None	5 (41,7%)	8 (38,1%)	13 (39,4%)
	Sharing a parent’s cellphone	6 (50,0%)	11 (52,4%)	17 (51,5%)
	Personal devices	1 (8,3%)	2 (9,5%)	3 (9,1%)
Tutoring	Yes	2 (16,7%)	4 (19,0%)	6 (18,2%)
	No	10 (83,3%)	17 (81,0%)	27 (81,8%)

All participants belonged to a single Grade V class of a public elementary school in urban Karawang, Indonesia. Informed consent was obtained in writing from parents or legal guardians before data collection. The study purpose, procedures, potential risks, and voluntary nature of participation including the right to withdraw at any time were clearly explained. Verbal assent was obtained from child participants in accordance with ethical guidelines appropriate for their age and developmental stage which aligns with ethical guidelines from the Declaration of Helsinki and the American Psychological Association (2017).

Participation was entirely voluntary, with no coercion or penalties for those who chose not to participate. During data collection, students could request clarification on assignments but were not involved in post-collection data validation due to the written nature of the narrative. To maintain confidentiality and anonymity, all participants were assigned pseudonyms (e.g., Student M1 for male student 1, student F1 for female student 1), and no personally identifiable information such as real names or exact locations was disclosed in the analysis or reporting.

### Data collection

The main instrument in this study was a narrative writing assignment. Students were asked to write a narrative story on the theme “Holiday Experience.” Instruction emphasized the development of a complete narrative structure, including an introduction, conflict, climax, and resolution. However, the focus was not only on technical quality but also on how the written narrative reflects self-representation, social relationships, and the influence of gender norms in their written expression.

The selection of the personal narrative assignment “Holiday Experience” was deliberate for several reasons. First, this theme is familiar and culturally relevant to Indonesian elementary school students, thus facilitating authentic self-expression and minimizing cultural bias. Second, for narrative evaluation, this study adopted the narrative assessment framework developed by Burhan Nurgiyantoro (an Indonesian linguist and literary expert), which is widely recognized in educational research in Indonesia. This instrument integrates narrative elements (plot, characterization, setting, point of view) with language use and writing conventions, enabling culturally sensitive and contextualized assessment of students’ narratives. The use of Nurgiyantoro’s framework ensures that the narrative analysis respects the linguistic and cultural narrative structures of Indonesia.

The categories for narrative analysis are detailed in Table 2, and cumulative scores across these categories yield a final grade for each student’s narrative writing. The following is a narrative writing assessment developed by ( Nurgiyantoro, 2015).

**Table 2.** Aspects of writing a narrative.

Aspects	Indicator					Scale *			
						1	2	3	4
Content (35%)	Plot								
	Characterization								
	Setting								
	Point of View								
Organization (25%)	Structure								
Language (20%)	Sentence Structure and Phrase Formation								
Grammar (15%)	Use of sentence structure, word/phrase arrangement								
Punctuation (5%)	Spelling and Punctuation								

* Description: 4 (Excellent), 3 (Good), 2 (Satisfactory), 1 (Poor).

The narrative writing assessment rubric is available at https://doi.org/10.6084/m9.figshare.31697821.

In addition to the narrative writing assignment, interviews were conducted with 10 purposefully selected students to gain deeper insight into their writing process and perceptions of gender norms. Interviews were recorded using a smartphone recorder and then transcribed verbatim. Transcripts were anonymized using pseudonyms to protect participants’ privacy. To ensure data accuracy, the transcriptions were double-checked and verified by the researcher through thorough rereading of the audio and transcripts. Interview data were then coded to identify emerging themes related to students’ experiences in narrative writing and their awareness of the influence of gender on their expression.

**Figure 1.**
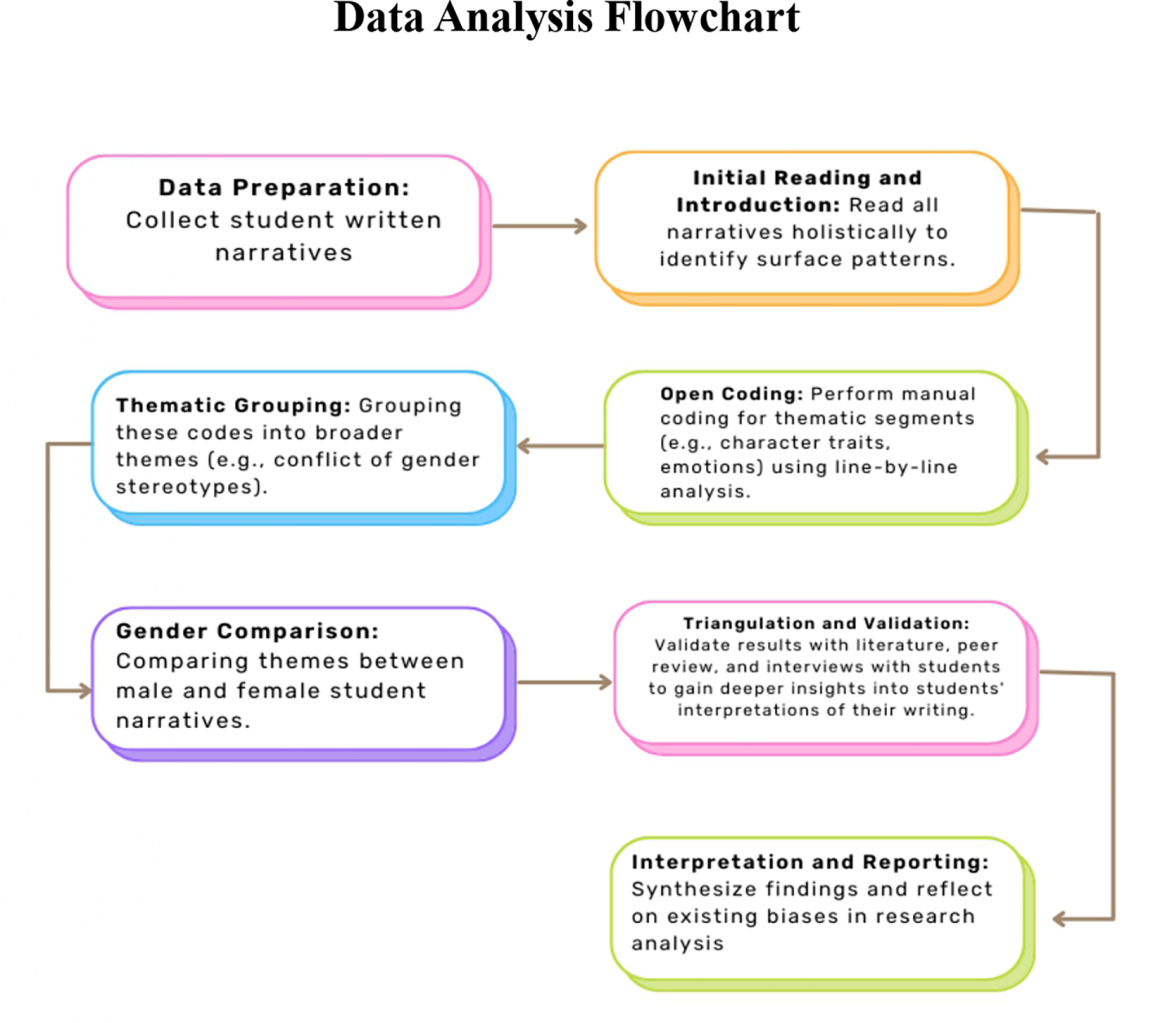
Data analysis flowchart. Figure 1 shows the analysis process following a systematic analysis using a critical thematic approach.

Figure 1 shows the analysis process following a systematic analysis using a critical thematic approach.

From the Table 2, the assessment of narrative writing ability using the formula:

$$\text{Value}=\frac{\text{Scores obtained in each aspect}\;}{\text{Maximum score of each aspect}}x\;\text{weight}\;\mathrm{on}\;\text{every aspect}$$

From the calculation of the formula above, the value in each aspect is cumulative so that the final value in writing a narrative can be obtained. This category was chosen not only as a technical tool but also as a means of reading the patterns of ideological expression implied in children’s writing.

The next data collection was conducted by researchers using interviews with 10 selected students to provide deeper insight into their narrative writing process. The data collection process involves recording and transcribing the results of the interview. Each interview is recorded using an audio recorder on a smartphone device. After the interview is over, the researcher transcribes the audio recordings to ensure that the data obtained can be analyzed carefully and accurately.

This transcription is then used to identify themes that emerge in students’ conversations about their experiences in writing narratives, as well as how they perceive gender norms in their writing. All transcripts are required to be anonymized to protect the privacy of the participants, using pseudonyms or identifier codes that do not reveal personal information such as the participant’s real name or location. The transcription process is carefully carried out to ensure that no information is missed or misinterpreted. After the transcription is complete, the researcher also verifies by re-reading the results of the interview and transcription to ensure the accuracy of the data collected.

### Data collection procedure

The data collection process was carried out for one week, from October 7 to October 12, 2024, with the task of writing a narrative given on October 3, 2024. Data collection was carried out by assigning narrative writing tasks to all students simultaneously in class for 60 minutes. Instruction is given orally and in writing to ensure students’ understanding of the assignment given. No changes were made to the planned procedures, as the original design had been adapted to the possible class conditions.

After the assignment is completed, the students’ writing is collected for further analysis. Handwriting is collected directly in the form of a physical document; there is no use of voice recorders or additional software during data collection. However, to obtain more in-depth data, interviews were conducted with 10 selected students to dig deeper into their experiences in writing narratives and their views on the influence of gender norms in their writing. Interviews are conducted in a structured manner with relevant questions to gain insight into the thinking process and students’ perspectives on research themes.

### Data analysis

The data analysis process follows systematic steps as listed below.

Data were analyzed using a critical thematic approach based on ( Braun & Clarke, 2006; Nowell, Norris, White, & Moules, 2017), which emphasizes identifying, analyzing, and reporting patterns (themes) in qualitative data while critically examining power structures such as gender norms that influence narratives.

The analysis process began with open coding of all student narrative texts and interview transcripts, conducted manually without software. Through repeated and iterative reading, text segments were highlighted and labeled to capture meaningful units representing key aspects of narrative content, structure, language use, and gender expression.

To enhance transparency and replicability, the coding process followed a clear coding guide that included:

Operational definitions for each coding category, such as:

For Physical Action Orientation, an example coding might be:

“Student 1: ‘I like to write about playing soccer or swimming because that’s what I often do.’”

Coding: Physical Action Orientation (A).

For Emotional/Relational Orientation, a coding example might look like this:

“Student 6: ‘I like writing about my vacation experiences with my family because it makes me feel happy.’”

Coding: Emotional/Relational Orientation (E).

Concrete examples of coded text excerpts illustrating each category are provided as supplementary materials.

Decision rules that clarify ambiguous cases ensure consistency in coding. After open coding, codes were grouped into higher-level themes reflecting narrative styles and gender-related literacy practices. Comparative thematic analysis was conducted to explore specific patterns in male and female students’ narratives, including the potential marginalization of certain forms of expression.

To ensure analytical reliability, two independent coders separately coded portions of the data, and inter-rater agreement was evaluated using Cohen’s Kappa coefficient.

Furthermore, quantitative data in the form of the final score of narrative writing is tested for normality with appropriate tests (e.g. Shapiro-Wilk) to ensure the readiness of the data in undergoing parametric statistical tests. The selection of parametic statistical tests, especially the Independent Samples T-Test, was used as an attempt to test whether there was a statistically significant difference between male and female narrative scores.

However, it should be emphasized that this quantitative analysis is not the main goal of the study, but rather serves as a complement that reinforces and confirms the findings of qualitative analysis that are more in-depth and rich in social context. Thus, quantitative data is seen as part of data triangulation to answer complex research questions related to the influence of gender and social values in literacy practice.

Data triangulation was conducted by comparing narrative and interview findings with existing literature on children’s narratives and gender roles, such as research by Clarke, Marks, & Lykins (2016); Odağ (2013); Radina (2019). Furthermore, collaborative reading and discussion between multiple researchers helped minimize subjective bias in data interpretation and increased the credibility of the results ( Lincoln & Guba, 1985). Discussions between researchers were conducted to minimize subjective bias in data interpretation.

All handwritten narratives were transcribed into password-protected digital files and stored on a secure university server with two-factor authentication and encrypted backups, in accordance with Indonesian personal data protection law ( *Law of the Republic of Indonesia Number 27 of 2022 on Personal Data Protection*, 2022). Anonymity was ensured by immediately coding the data to de-identify and deleting copies of the raw files after analysis.

### Ethical consideration

The research and community service institute of Universitas Buana Perjuangan Karawang has approved this research with the contract number: 062/LPPM/UBP/2025. The researcher gave a letter of approval has also been given by the researcher to all respondents. Written consent to participate from the respondent was obtained in accordance with document 400.3.5/110/SD/2025. Respondents gave their consent without force from anyone. Subsequently, in order to protect the rights and privacy of the respondents, all forms of data acquired will remain confidential.

## Results

This study aimed to explore how gender norms and social values influence the narrative writing styles of male and female elementary school students, and the implications of these differences for inclusive literacy education. The analyses address the following sub-questions: differences in narrative content, organization and cohesion, language use and diction, technical writing errors, and pedagogical implications.

Reliability of narrative assessment was supported by a Cohen’s Kappa value of 0.561 (p < 0.001), indicating moderate and statistically significant agreement between the two independent assessors. This strengthens the credibility of the scoring process (see Table 3 for details).

**Table 3.** Reliability of narrative assessment. Increase the reliability of the results by using Cohen’s kappa.

Measure of agreement	Kappa	Asymptotic standard error ^a^	Approximate T ^b^	Approximate significance
Measure of Agreement	0.434	0.087	13.291	0.000
N of Valid Cases	33			

^a^ Not assuming the null hypothesis.

^b^ Using the asymptotic standard error assuming the null hypothesis.

This visualization is based on the dataset available at https://doi.org/10.6084/m9.figshare.30281671.

Based on the Kappa analysis results presented in the Table 3, a Kappa value of 0.434 indicates a moderate level of agreement between raters in assessing male and female students’ narrative writing. This value indicates that there is sufficient agreement between raters, but there is still room for improvement in terms of agreement. A p-value of 0.000 indicates that this result is highly statistically significant, meaning that the agreement recorded is not a coincidence and reflects true agreement between raters. Overall, despite some differences in the ratings, the recorded agreement indicates that the ratings are valid and reliable in the context of this study.

Furthermore, this study used a normality test to determine whether the final narrative writing scores followed a normal distribution, allowing for parametric statistical analysis. The results of the normality test are presented in Table 4 above.

Based on the results of the normality test using Shapiro-Wilk, it is known that the final score data of students’ narrative writing skills is distributed normally, both in the male group (Sig. = 0.825 > 0.05) and the female group (Sig. = 0.521 > 0.05), thus meeting the assumption to conduct a parametric test. With this assumption of normality met, the difference in average final scores between male and female students can be further tested using the Independent Samples T-Test to determine whether the achievement gaps identified in the descriptive analysis constitute a statistically significant difference.

**Table 4.** Normality Test.

Tests of normality						
Gender	Kolmogorov-Smirnov ^a^			Shapiro-Wilk		
	Statistic	df	Sig.	Statistic	df	Sig.
Final Score						
1	.128	12	.200 *	.963	12	.825
2	.137	21	.200 *	.960	21	.521

* This is a lower bound of the true significance.

^a^ Lilliefors Significance Correction.

This study also calculated a quantitative comparison of the final narrative writing scores between male and female students with the aim of determining whether there is a statistically significant difference in overall narrative writing performance between the two genders. The following are the results of the t-test calculation presented in Table 5.

**Table 5.** Independent samples T-Test test results final score writing narrative.

Levene’s test for equality of variances		t-test for equality of means						95% Confidence interval of the difference		
		F	Sig.	t	df	Sig. (2-tailed)	Mean Difference	Std. Error Difference	Lower	Upper
Final Score	Equal variances assumed	.506	.482	−1.288	31	.207	−6.250	4.853	−16.147	3.647
	Equal variances not assumed			−1.343	25.983	.191	−6.250	4.655	−15.819	3.319

Based on the results of the independent sample t-test shown in Table 5, there is no statistically significant difference in the final scores between male and female students [t(31) = −1.288, p = 0.207]. Homogeneity of variance is confirmed (Levene’s test p = 0.482). The 95% confidence interval for the difference includes zero, which confirms that the observed mean difference (6.25 points) is not statistically significant.

Students’ narrative writing is analyzed not only based on technical structure but also as a reflection of self-representation, social relationships, and gender norms that shape their mindset in expressing experiences. The narrative writing scores were obtained from 33 students whose assessments were based on the following aspects of narrative writing (see Table 6 for details).

**Table 6.** Narrative writing scores based on student narrative writing.

Aspects of narrative writing		Narrative writing of male student		x̄ Male	Narrative Writing of Female Students		x̄ Female	
		Assessor 1	Assessor 2		Assessor 1	Assessor 2		
Content (35%)	x̄ Plot	2,9	2,3	2,6	3	3,1	3.0	
	x̄ Characterization	2,7	2,2	2,45	2,4	2,1	2.0	
	x̄ Setting	2,7	2,4	2,55	2,8	3	2.7	
	x̄ Point of View	3,7	3,8	3,75	3,8	3.8	3.8	
	**x̄ Content**	**27,8**	**23**	**26**	**24,5**	**26,1**	**25,3**	
Organization (25%)	x̄ Structure	2,3	2,5	2,4	3.0	3	3.0	
	**x̄ Organization**	**14,6**	**15,6**	15,1	**18,4**	**18,4**	**18,4**	
Language (20%)	x̄ Sentence Structure and Phrase Formation	2,3	2,7	2,5	2,5	2,5	2.5	
	**x̄ Language**	**11,7**	**13,3**	**12,5**	**12,6**	**12,6**	**12,6**	
Grammar 15%	x̄ Grammar	1,8	2,5	2,15	2,5	2,8	2.6	
	**x̄ Grammar**	**6,9**	**9,4**	**8,15**	**9,5**	**10,4**	**9,95**	
Writing Conventions (10%)	x̄ Spelling and Punctuation	1,7	1,8	1,75	2,3	2	2.0	
	**x̄ Writing Conventions**	**2,1**	**2,3**	**2,2**	**2,9**	**2,5**	**2,7**	
	**x̄ Final Score**	**63,1**	**63,6**	**63,95**	**67,9**	**70**	**68,95**	

*Note:* This visualization is based on the dataset available at https://doi.org/10.6084/m9.figshare.30281671.

Based on the Table 6, it can be seen that the average final narrative writing score of female students (68.95) is higher than that of male students (63.95). This difference indicates that female students have better narrative writing performance overall. Although male students scored slightly higher on the content aspect (26 compared to 25.3), female students excelled in the aspects of organization (18.4 compared to 15.1), grammar (9.95 compared to 8.15), and writing conventions (2.7 compared to 2.2), and were relatively equal in the language aspect (12.6 compared to 12.5). Thus, female students’ superiority lies primarily in their ability to compose writing in a more structured manner, use more appropriate grammar, and apply writing conventions better.

### Story content

**Figure 2.**
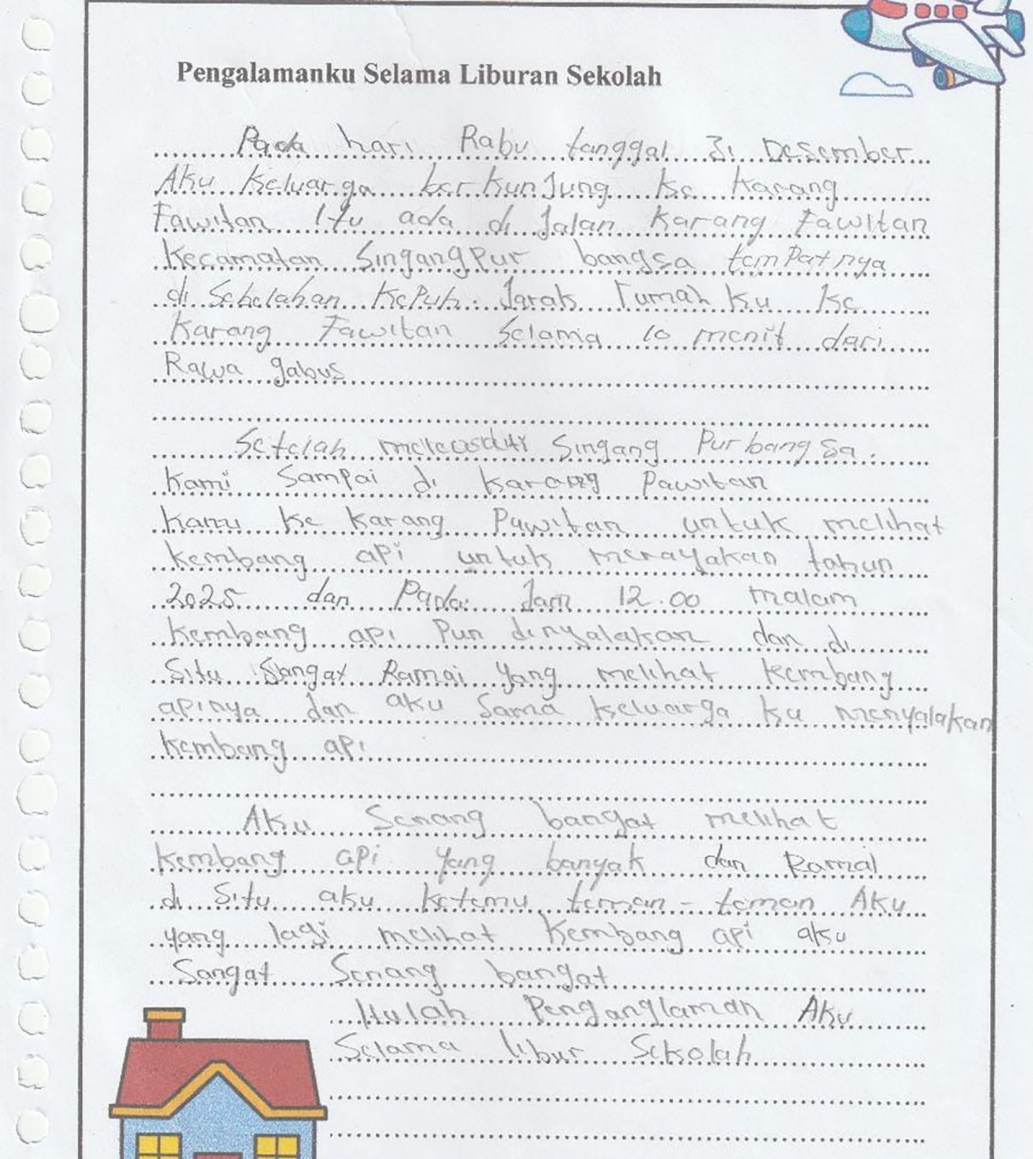
Male student narrative writing results (This visualization is based on the dataset available at https://doi.org/10.6084/m9.figshare.30353890.v1).

Male student writing tends to feature an action-oriented narrative, with a focus on physical activities such as playing ball, swimming, and everyday activities ( Anggraeni, Sunendar, & Cahyani, 2025a). Their stories show a linear, simple plot with a plot score of 2.4 and often without conflict or strong resolution. Characters in the story are only mentioned without character development or deep emotional expression, which is reflected in the characterization score of 2.0. The following is an example of the results of the narration writing of male students shown in Figure 2.

The writing of male students shows a tendency to construct stories based on repetitive and linear series of physical activities, as in the quote, “My sister and I were swimming. My sister and I played on slides. My sister and I bought sausages…” This plot does not show any climax, conflict, or emotional reflection. The narrative stops at reporting activities without depth of meaning. This reinforces the view that male narrative styles are formed to show ‘liveliness,’ not ‘feelings.’ The setting is used only as an action support, with no depiction of the atmosphere, which is reflected in the setting score of 2,5. This is a common pattern of male narratives that do not place value on an emotional or atmospheric setting. In addition, the narrative of male students shows that the representation of the figure of ‘me’ is only portrayed as a perpetrator of the activity, without reflection of feelings or relational involvement. This reflects the form of narrative shaped by masculinity norms that emphasize independence, physical strength, and minimal emotional expression. One of the male students stated in an interview: *“I prefer to write about playing football or swimming because that’s what I do the most. Usually, I just tell what happened without thinking too much about the feelings or relationships between the characters. That’s not really important in my opinion.” *( Anggraeni, Sunendar, & Cahyani, 2025b).

**Figure 3.**
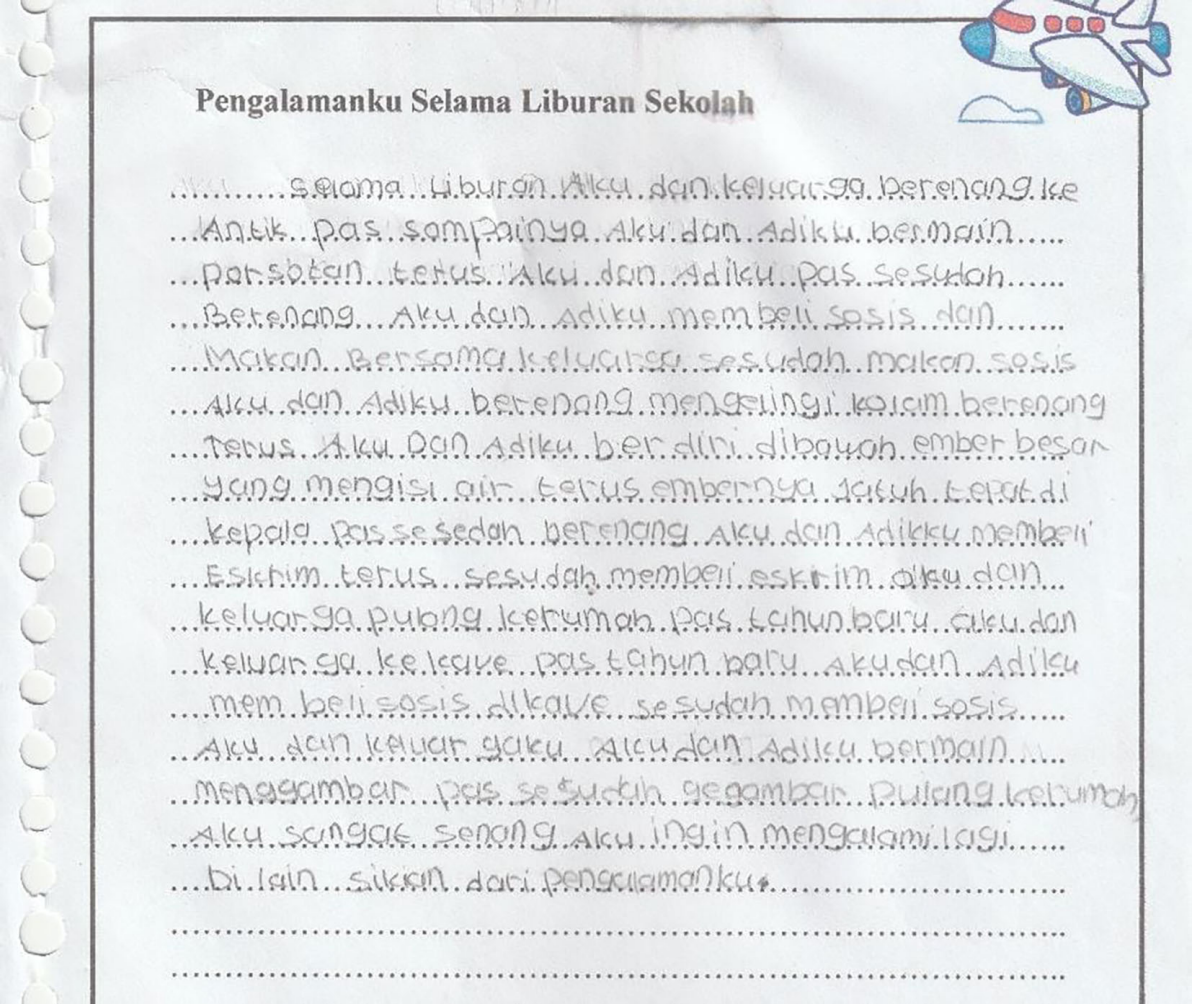
Results of narrative writing for female students (This visualization is based on the dataset available at https://doi.org/10.6084/m9.figshare.30353890.v1).

In contrast, female students’ narratives show a tendency toward emotional expression, depiction of atmosphere, and deeper interpersonal relationships. The main character not only performs activities but also establishes social relationships and portrays feelings in certain situations. This shows that self-expression in their writing is more influenced by affective values and feminine norms such as empathy and attachment ( Anggraeni et al., 2025a). As one female student stated: *“I love writing about my vacation experiences with my family because it makes me feel happy.”* ( Anggraeni et al., 2025b) *.* This is supported by a higher narrative content score of female students, which is 28.7 compared to male students, who are 26.5. The following is an example of the narrative writing of female students shown in Figure 3.

The narrative writing of female students in Figure 3 can build a plot with a time and emotion orientation. This is reflected in the plot score of 3.0 and the setting score of 2.7. It exhibits a reflective and expressive narrative style, which is culturally more attached to the feminine role: organizing, paying attention to the atmosphere, and expressing feelings. As in the excerpt of the story piece “On Wednesday, December 31st… We went to Karang Pawitan to see the fireworks to welcome 2025… I’m so happy to see so many fireworks…”

Thus, female students excelled in content, although male students scored slightly higher on characterization (2.45 vs. 2.0), indicating that male narratives may place greater emphasis on character or figures in their stories. The implications of this difference suggest that gender norms and social values contribute to the different focuses of narrative content, with female students tending to adopt feminine norms that emphasize social connections and emotional details through the use of richer settings and plots, while male students emphasize more active characters and roles, in line with masculine norms ( Anggraeni et al., 2025a).

### Organization

The writing of male students shows a fragmented narrative structure, with jumps between activities without strong logical transitions. Coherence between sections is often broken, reflecting event-oriented narrative processing rather than reflection or character development. This is reflected in the score of 15.1, which is lower than that of the female student. This can be understood as part of a ‘masculine’ narrative construction, which prioritizes action over relationships. As in the phrases “Me and my sister swim…” “My sister and I bought sausages…” and “My sister and I play…” There is a lack of structural variation; it tends to be mechanical. Sentences do not develop semantically. This style is often considered ‘less attractive’ academically, but it may reflect a more “straight to the core” male communication pattern that is unfortunately often not valued in literacy curricula.

This finding is supported by interview data, where one male student stated:

*“I don’t really like writing complicated stories. I prefer a story that is fast and straight to the point. If something is interesting, I write it straight away without much thought about how the story should proceed.” *( Anggraeni et al., 2025b) *.*

This statement reinforces the idea that fragmented structure is not simply a technical weakness but also a deliberate narrative choice aligned with a preference for brevity and directness.

Meanwhile, female students demonstrated the ability to organize narratives in a more organized manner, although some stories still ended abruptly. This is supported by their higher essay structure score of 18.4. The cohesion of ideas and emotions is more evident in their narratives, showing an awareness of the need to unite social and mental experiences in a single story. In the narrative of the female, there are enough connectors of ideas (“after that,” “we got to …”) indicating an effort to construct a cohesive narrative despite its technical imperfections. This emphasis on the continuity of events and emotions is characteristic of narratives sensitive to social relationships and feelings, in contrast to the often disjointed and abrupt topic changes of masculine narrative styles.

Interview evidence supports this interpretation. One female student explained:

*“When writing stories, I always try to make sure that all parts of the story are well connected. I think a good story should have a clear plot and describe how the characters in each section feel.”* ( Anggraeni et al., 2025b) *.*

Thus, female students’ narrative organization skills are better than male students’. This reflects the social value of gender that values relationships and regularity of expression; this presents a challenge for educational practices to support and appreciate the more direct or fragmented narrative styles of males without losing the diversity of expression.

### Language

The male’s writing has short sentence structures, simple word choices, and a lack of emotional variation. This is reflected in his language use score of 12,5, indicating concise, action-oriented communication. For example, in the boy’s narrative example in Figure 2, there are many repetitions of words, for example, the words “swimming,” “playing,” and “me and my sister.” This shows that the sentence structure is very simple. Words are chosen based on actions, not reflections or expressions. This shows the role of gender stereotypes in language choices, where boys tend to avoid emotional expressions that are considered feminine.

This interpretation is supported by interview data. One male student expressed:

*“I usually write simple words. When I write my feelings, I am confused about how to write them. So I’m just telling you what I did.” *( Anggraeni et al., 2025b) *.*

This statement indicates that boys’ preference for simple, repetitive diction is not only a technical limitation but also a reflection of discomfort with emotional vocabulary, aligning with the masculine norm of emotional restraint.

In contrast, female students tend to use more expressive diction, emotional verbs, and sentence formulation that contains evaluations of situations. This is supported by their slightly higher language score of 12.6, with a tendency to use expressive words such as “I’m really happy …” and “very crowded …” This diction is simple, yet it attempts to convey mood. Female students’ narrative writing uses more expressive and repetitive words, indicating that this writing is directed at expressing emotion and experiential involvement, rather than simply recording actions. This supports the finding that female narratives emphasize subjective experience and emotional engagement, something less evident in male students’ writing.

The interviews with female students corroborate this finding. One female student explained:

*“When I write stories, I like to write what I feel so that the reader knows I’m happy or sad. So I choose words that can show feelings.”* ( Anggraeni et al., 2025b) *.*

This response shows that female students have greater intentionality in choosing words that evoke feelings and atmosphere, reinforcing their higher scores in narrative expressiveness.

In the aspect of the use of language in narrative writing, the scores of male and female students showed almost the same scores, with an average of 12.5 for boys and 12.6 for girls. This reflects that both groups have a balanced ability to form sentences and phrases technically. Both male and female students use quite good sentence structure and phrase formation, so there are no significant differences in general language aspects. Thus, these findings suggest that language skills as a technical aspect are not the main factor that differentiates narrative writing styles between men and women.

### Grammar

The grammar mastery of female students showed a significant advantage over male students, with an average score of 9,95 compared to 8,15. This illustrates that female students have better grammatical skills in writing narratives. This superior grammar ability can be caused by a more careful attitude in the use of language as well as the existence of social values that encourage perfection in written expression.

Women’s superiority in this aspect of grammar also supports a social perception that associates women with the traits of “prudence” and “attention to detail.” These traits are often considered part of the feminine gender role in cultural contexts, which is then reflected in the quality of their writing. In other words, social norms and gender constructs not only shape the content and style of the narrative, but also influence the technical and neat aspects of student writing.

### Writing conventions

The writing conventions aspect which includes spelling and punctuation shows that female students have an average score of 2.7, higher than male students who have an average score of 2.2. This difference illustrates that female students are generally more careful in adhering to technical rules of grammar, such as punctuation placement and spelling accuracy. This rigor contributes to the level of professionalism and ease of reading the narratives written by female students, so that their narratives are not only content rich but also easy to understand and easy to read.

Such greater precision in these aspects of technical writing may also reflect social norms and gender roles that foster greater attention to detail and perfection in women’s writing. Meanwhile, male students may give less priority to this technical aspect, which can lead to more spelling mistakes or punctuation in their narratives.

### The Influence of Socio-Economic Status (SES) on Final Scores

As explained in the participant section, students in this study come from socio-economic backgrounds dominated by the lower middle class. To further understand how SES factors contribute to the quality of narrative writing, a Pearson correlation analysis was conducted between nine SES indicators and students’ final scores. This analysis is important to identify the factors that have the most influence on students’ writing achievement, as well as to be the basis for formulating targeted pedagogical recommendations. Table 7 presents the results of the correlation analysis.

**Table 7.** Correlation between SES indicators and final values.

Indicator SES	Correlation Coefficient (r) with Final Score	Significance (p)	N	Interpretasi
Father’s Education	0,784**	0,000	33	Strong, significant
Mother’s Education	0,837**	0,000	33	Very powerful, significant
Father’s Work	0,778**	0,000	33	Strong, significant
Mother’s Work	0,614**	0,000	33	Moderate, significant
Number of Books	0,773**	0,000	33	Strong, significant
Reading Activities	0,856**	0,000	33	Very powerful, significant
Parent Assistance	0,771**	0,000	33	Strong, significant
Internet Access	0,710**	0,000	33	Strong, significant
Tutoring	0,410*	0,018	33	Moderate, significant

Description: ** = p < 0.01; * = p < 0.05.

This visualization is based on the dataset available at https://doi.org/10.6084/m9.figshare.31653961 ( Anggraeni et al., 2026a).

The results of Pearson’s correlation analysis showed that all socio-economic status indicators (SES) had a significant positive relationship with the final score of students’ narrative writing. **Reading activity** (r = 0.856; p < 0.01) and **maternal education** (r = 0.837; p < 0.01) showed the strongest correlation, falling into the very strong category. This indicates that active reading habits and mother’s educational background are the factors most closely related to the quality of students’ narrative writing.

The indicator groups with strong correlations included **father’s education** (r = 0.784), **father’s work** (r = 0.778), **number of books at home** (r = 0.773), **parental assistance** (r = 0.773), and **internet access** (r = 0.771). These five indicators have relatively equal correlation strength, showing that home environmental factors are interrelated in shaping children’s literacy ecosystems.

Meanwhile, **maternal work** (r = 0.614) and **tutoring** (r = 0.444) showed a moderate correlation. Interestingly, maternal education correlates much more strongly than maternal employment, indicating that *maternal knowledge and capabilities* are more important than *economic status* alone. Similarly, the activity of reading is stronger than just the possession of a book, confirming the importance of *the quality of interaction* with the reading material.

### Gender norms and stereotypes in narrative writing

Based on the study of the results of narrative writing, it reveals how gender norms and stereotypes are implicitly represented and reproduced in student writing. The narrative of male students tends to assert traditional masculinity norms that emphasize independence, physical activeness, and emotional control. The ‘me’ figure in male narratives is more often portrayed as an actor without deep emotional involvement, which reflects social expectations for men to appear strong and not affectively expressive ( Anggraeni et al., 2025a).

This interpretation is reinforced by interview data. One male student stated:

*“I rarely write about feelings because I think it’s weird. It’s better for me to tell my story about what I do, like play ball or go swimming.” *( Anggraeni et al., 2025b) *.*

This quote illustrates how masculine identity construction leads boys to avoid affective expressions and focus on actions, aligning with hegemonic masculinity norms that value independence and physical activity.

In contrast, female student narratives feature feminine norms that emphasize social attachment, empathy, and emotional expression. Characters in women’s narratives not only play an active role but also show a connection with others and a rich reflection of feelings.

A female student shared during the interview:

*“I like to write stories that have a feeling of sadness or joy. If there are no feelings, the story is like a lack of life.” *( Anggraeni et al., 2025b) *.*

This statement confirms that girls see emotions as an integral part of storytelling, reflecting cultural expectations that validate empathy and relational sensitivity.

These findings suggest that even though students write based on personal experiences, they are still influenced by the social and cultural constructs that shape their understanding of gender roles. The interviews demonstrate that boys’ avoidance of emotional vocabulary and girls’ preference for emotive and relational narratives are not purely individual tendencies but are socially reinforced patterns.

### Gendered narrative styles

The comparison between the narratives of male and female students shows a different pattern of dominance of expression, where the male narrative style is more oriented towards external actions and events, while the female narrative style emphasizes interpersonal relationships and emotional depth. This pattern reflects not only individual preferences but also the potential marginalization of certain styles of expression in educational contexts. For example, more direct and simple male narratives are often undervalued in literacy assessments that prioritize the emotional depth and complexity of the narrative, which tend to be associated with female styles ( Anggraeni et al., 2025a).

Interview data provides additional insight into this phenomenon. One male student stated:

*“I like stories that end quickly, like telling me to play soccer, then go home. If I have to write long and have a lot of feelings, I get lazy.” *( Anggraeni et al., 2025b) *.*

This quote highlights that brevity and action-focus are conscious narrative choices rather than merely technical limitations, suggesting that boys value efficiency and directness in their storytelling.

Conversely, a female student shared:

*“I love that my story can make others feel what I feel. So I wrote it down so that the reader can imagine the atmosphere.”* ( Anggraeni et al., 2025b).

This illustrates that girls intentionally craft narratives to evoke empathy and emotional engagement from readers, which aligns with their higher scores in content and organization.

This pattern has implications for the reproduction of gender inequality in literacy education, where masculine expressions can be marginalized or considered less valuable when assessment criteria favor detailed, emotionally rich narratives. These findings are in line with critical literacy theory that emphasizes the importance of critiquing hidden norms and values in literacy practice, as well as gender studies that show how education can reproduce or challenge gender stereotypes.

## Discussion

The novelty of this research lies in an integrated approach to analyzing the narrative writing of elementary school students in the context of Indonesian education, combining quantitative assessments from multiple learners with qualitative thematic analysis to explore how gender norms and stereotypes manifest in children’s self-representation, social relationships, and emotional expression. Unlike previous studies that focused on older populations especially in Western contexts (e.g., Grysman, 2018; Odağ, 2013). This study highlights how gender narrative styles emerge in young Indonesian students (ages 10–12 years), who are influenced by culturally specific understandings of masculinity and femininity.

Before examining gender differences, this study first considered the potential influence of socio-economic status (SES) on students’ narrative writing quality. As presented in Table 7, Pearson correlation analysis revealed that all SES indicators had significant positive relationships with final writing scores. Reading activities and mother’s education showed the strongest correlations, categorized as very strong. These were followed by father’s education, father’s occupation, number of books at home, parental assistance, and internet access, all showing strong correlations. Mother’s occupation and tutoring showed moderate correlations. Importantly, the finding that gender differences in writing styles remained consistent across SES levels (as discussed in subsequent sections) suggests that gender norms have an independent influence on literacy development, distinct from socio-economic factors. Although socioeconomic status (SES) influences access to literacy resources, the quality of parent-child interactions and cultural practices surrounding literacy may be equally or even more influential ( Nag, 2023; Neumann, 2016). The particularly strong correlation between mother’s education and children’s writing outcomes supports extensive literature on the intergenerational transmission of literacy ( Telias et al., 2022), while the relatively weaker correlation for tutoring suggests that formal external interventions may be less effective than home-based literacy environments.

Regarding narrative content, male students’ writing tends to emphasize physical and everyday activities, such as playing ball or swimming, with a straightforward plot, limited climax, and relatively superficial characterization. The narratives often privilege action over emotional depth or interpersonal relationships. This pattern is consistent with the concept of hegemonic masculinity, which highlights action, physical dominance, and the suppression of emotional expression as markers of masculine identity ( Bartholomaeus, 2012; Jewkes et al., 2015). In the context of primary education, such social norms may encourage boys to develop a linear and action-oriented narrative style while distancing them from expressions of affection and social reflection ( Duncanson, 2015),As s a result, this narrative tendency may overlook the emotional dimension that is important for the development of more complex narratives ( Bradford Wainwright, 2019). For example, in a family vacation story with no clear resolution, the narrative focuses mainly on physical events without providing space for emotional reflection. This tendency reflects a masculine habitus that prioritizes physical independence and action dominance in the construction of gender identity ( Elliott, Roberts, Ralph, Robards, & Savic, 2022; Sims-Gould et al., 2018). It may also indicate how emotional expression is often marginalized because it is associated with weakness, which contrasts with dominant constructions of masculinity that begin to take shape from an early age ( Goldstein, 2003). By contrast, female students’ narratives tend to contain richer emotional expression, more detailed settings, and stronger interpersonal relationships, suggesting a greater orientation toward empathy and social connectedness. Although these observations resonate with ideas such as hegemonic masculinity and feminine scripts, the qualitative interview data remain limited in fully supporting adult sociological frameworks; therefore, these interpretations should be presented cautiously to avoid overgeneralization ( Fernández & Sartori, 2012).

In addition, male students’ narrative writing often lacks a clear climax or resolution, suggesting a tendency to organize stories as a linear sequence of events rather than as a progression of conflict. This pattern may reflect greater engagement with action-oriented texts and simpler linear structures with limited reflective depth ( Odağ, 2013). Moreover, the socialization of masculine norms may contribute to the development of narratives that are chronological and less emotionally elaborated ( Radina, 2019). From the perspective of narrative theory, such texts tend to lose the elements of transition and dynamics, which are essential markers of a complete narrative ( Simon-Shoshan, 2013). Comparative studies show that men are more involved with action-oriented texts, while women with action-oriented texts are inward-experiential ( Odağ, 2013). Such differences may be associated with women’s relatively higher empathy and emotional intelligence ( Clarke, Marks, & Lykins, 2016), as well as with social patterns that more openly permit the expression of affection and emotion ( Walton, Coyle, & Lyons, 2004). In this study, female students likewise tended to produce narratives with greater coherence and emotional depth, although this tendency should be interpreted carefully given the limited sample and variability across participants.

Male narratives often lack a clear climax or resolution and follow a linear sequence of events ( Odağ, 2013), consistent with the socialization process that encourages emotional restraint in boys ( Radina, 2019). From the perspective of narrative theory, this kind of text tends to lose elements of transition and dynamics, which are important markers of a complete narrative ( Simon-Shoshan, 2013). In contrast, women’s narratives show thematic coherence and greater emotional depth ( Grysman, 2018; Schulkind, Schoppel, & Scheiderer, 2012), in accordance with the findings of comparative studies showing that women are more engaged with inward-oriented experiences-oriented texts, while men are more focused on Action-oriented texts ( Odağ, 2013). The strong correlation between reading activities and writing quality found in this study reinforces the importance of literacy practices in shaping these narrative capabilities, with female students reporting higher reading frequency compared to male students, as shown in Table 1. This difference is also related to higher empathy and emotional intelligence scores in women ( Clarke, Marks, & Lykins, 2016), as well as social patterns that allow women to express affection more openly ( Walton, Coyle, & Lyons, 2004). However, the variability in the group and the limited sample size suggest that this pattern is a trend, not an absolute law.

Another difference lies in the cohesion and organization of the story. In narrative organizations, male students tend to produce fragmented stories with fewer logical connections, reflecting a communication style that prioritizes brevity and action ( Schipke, 2005; Francis et al., 2003). Weaknesses in the use of these cohesion devices are also found in general in male students, which reinforces the view that this “jump-hop” style is the result of the interaction between gender norms and technical skills ( Albelihi, 2022). Nevertheless, this description of the style needs to be understood in the context of sample culture and education, and cannot be generalized as a universal trait.

Both groups exhibited language errors; however, the use of simple, repetitive diction by men may be partly due to masculine norms that inhibit emotional expression ( Allan, 2016; McQueen, 2017). The greater use of affective language of female students is aligned with cultural validation of female expressiveness ( Bohanek, Fivush, & Walker, 2005; Nureldeen, Alsabatin, Eskander, & Nasr, 2023). However, this language pattern is subtle and requires careful interpretation given the participant’s developmental stage.

Spelling and punctuation errors not only disrupt the clarity of the writing but also directly affect the way readers assess competence and trust in the author. ( Johnson, Wilson, & Roscoe, 2017). The greater frequency of errors in male narratives, perhaps due to shorter and more abrupt sentences, suggests that the mechanical aspects of writing disproportionately affect perceptions of their work regardless of their content. Additionally Witchel et al. (2020, 2022) shows that misspellings and improper capitalization lower readers’ trust in information, emphasizing the importance of mechanical aspects of writing in shaping readers’ judgments.

The observed gender differences reflect a broader socially constructed script of “men act, women feel,” which is perpetuated through family socialization and educational practices including assessment rubrics and exemplary texts. The correlation findings add a crucial dimension to this understanding: while gender norms shape narrative styles, these effects are embedded within broader socio-economic contexts. The strong correlations between home literacy indicators (books, reading activities, parental assistance) and writing outcomes suggest that interventions targeting the home literacy environment could benefit both genders, while the persistent gender differences even after controlling for SES (as evidenced by the consistent patterns across SES levels) indicate that gender-specific pedagogical approaches are also needed. This reinforces masculine expression by default and can marginalize or underestimate male narrative styles that favor conciseness over elaboration. Such dynamics create a paradox in which both genders can be limited in authentic narrative expression.

Overcoming these challenges requires a fair pedagogical response. Teachers can apply two-dimensional rubrics that separate technical accuracy from expressive content, allowing for the recognition of diverse narrative forces. Introducing gender crosswriting activities can encourage students to experiment outside of traditional forms of gender narratives. In addition, providing varied models of genres and styles broadens students’ literacy horizons. Critical reflection on educator bias in feedback is essential to avoid perpetuating narrow gender norms. These strategies foster a classroom environment that challenges stereotypes and supports the development of inclusive literacy.

## Conclusion

The analysis shows that there are differences in narrative writing styles between male and female students that are influenced by gender norms and social values. Female students tend to have a narrative with a more complex plot (score 3.0) and richer setting (score 2.7), as well as better organizational skills (score 18.4) than male students. In addition, women also excelled in grammar (score 9.95) and writing conventions (score 2.7), which showed greater precision and accuracy in the technical aspects of writing. However, in the characterization aspect, male students showed a slightly higher score (2.45), indicating that they were more emphatic about the portrayal of characters in their narratives. Overall, the final narrative score of female students (68.95) was higher than that of male students (63.95), although this difference was not too large.

These differences in writing styles reflect how masculinity and femininity norms direct students’ literacy expression at the elementary school level. Female students prioritize social connections, emotional depth, and coherent narrative structure, while male students focus more on action and character as the center of their story. The implication of these findings is the importance of implementing gender-sensitive literacy learning and assessment strategies that can accommodate diverse writing styles and help reduce gender stereotypes in educational practice to create an inclusive and equitable literacy environment for all students.

### Limitations and further research

This study has several limitations that should be acknowledged. First, the research was conducted in a single public elementary school in an urban setting, which may limit the generalizability of findings to rural schools or different socio-economic contexts. Second, the qualitative data were based on written narratives and interviews from a relatively small sample (33 students, 10 interviews), which, while sufficient for in-depth thematic analysis, may not capture the full diversity of narrative styles across Indonesia.

Future research should expand to multiple schools with diverse cultural and socio-economic backgrounds, employ larger and more balanced samples, and consider longitudinal designs to observe how gendered narrative styles evolve over time. Mixed-method approaches integrating quantitative linguistic analysis tools (e.g., corpus linguistics software) could provide additional objectivity. It would also be valuable to explore teacher perceptions and grading rubrics to determine how assessment practices directly shape and potentially reinforce these gendered narrative patterns. Finally, cross-cultural studies comparing Indonesian data with other countries in Southeast Asia could offer insights into whether these patterns are culturally specific or universal.

## Data Availability

- Figshare: ‘Analysis of Thematic Coding Results Based on Interviews’ Doi: https://doi.org/10.6084/m9.figshare.30283300 ( Anggraeni et al., 2025a). which contains anonymized thematic coding and interview data necessary for replicating the qualitative analysis. - Figshare: ‘Interview Results of Gender and Narrative Writing’ Doi: https://doi.org/10.6084/m9. figshare.30281446.v2 ( Anggraeni et al., 2025b), including transcribed and anonymized interview excerpts for themes related to gender norms. - Figshare: ‘Results of Observations of Male and Female Students’ Narrative Writing:’ Doi: https://doi.org/10.6084/m9.figshare.30281671 ( Anggraeni et al., 2025d), which provides observation data. - Figshare: ‘Narrative Writing by Male and Female Students’ Doi: https://doi.org/10.6084/m9.figshare.30353890.v1( Anggraeni et al., 2025c), which includes samples and figures for analysis, such as narrative points and themes. • Figshare: ‘Research data on the influence of gender and socio-economic status’ Doi: https://doi.org/10.6084/m9.figshare.31653961 ( Anggraeni et al., 2026a) • Figshare: ‘Rubric Instrument for Assessment of Narrative Writing Ability’ Doi: https://doi.org/10.6084/m9.figshare.31697821 ( Anggraeni et al., 2026b)
