# Supplementary material for: Gender and Narrative Writing in Elementary Schools: A Qualitative Study Supporting Inclusive Education
Source: F1000Res. 2026 May 2;14:1317. Originally published 2025 Nov 26. [Version 2] doi: 10.12688/f1000research.171604.2 (PMC13282602; doi:10.12688/f1000research.171604.2)
Supplement: Peer review history

# Reviewer report: Reviewer response for version 2

**DOI:** 10.5256/f1000research.195218.r481980

**Published:** 2026-06-19

**Authors:** Fajar Nugraha

**Affiliations:** 1 Universitas Garut, Langensari, Indonesia

Based on my review of this journal article, my primary concern lies in the research methodology employed. The data analysis used in this study appears to be more aligned with a qualitative research approach; therefore, consistency between the research design, data collection techniques, and analytical methods is essential. However, the study only uses a questionnaire as its research instrument. Relying on a single instrument may lead to less comprehensive findings, making the research results less adequate in capturing the phenomenon in depth. In addition, questionnaires are generally more associated with quantitative research due to their structured nature in measuring responses. Therefore, to improve the validity and credibility of the research findings, the author should consider incorporating supplementary instruments, such as interviews, observations, or documentation. Furthermore, we recommend using triangulation to cross-check and verify data from multiple sources or methods, which will help ensure more accurate, in-depth, and scientifically accountable results.

Is the work clearly and accurately presented and does it cite the current literature?

Yes

If applicable, is the statistical analysis and its interpretation appropriate?

Yes

Are all the source data underlying the results available to ensure full reproducibility?

Yes

Is the study design appropriate and is the work technically sound?

Yes

Are the conclusions drawn adequately supported by the results?

Yes

Are sufficient details of methods and analysis provided to allow replication by others?

Yes

Reviewer Expertise:

NA

I confirm that I have read this submission and believe that I have an appropriate level of expertise to confirm that it is of an acceptable scientific standard.

# Reviewer report: Reviewer response for version 2

**DOI:** 10.5256/f1000research.195218.r492692

**Published:** 2026-06-12

**Authors:** Liyana Sunanto

**Affiliations:** 1 Universitas Darul Ma'arif Indramayu, West Java, Indonesia

This article presents a timely and thoughtfully executed study on gender differences in narrative writing among Indonesian primary school students. The mixed-methods design, combining qualitative thematic analysis with quantitative t-tests and correlations, is appropriate and well-integrated. The authors demonstrate intellectual honesty by acknowledging methodological limitations—including moderate inter-rater reliability and an unbalanced gender sample—without overstating their findings.

 What makes this work particularly valuable is its grounding in real classroom contexts. The use of handwritten narratives, parent questionnaires, and student interviews lends authenticity, while the statistical patterns (e.g., female students excelling in organization and grammar; male students showing comparable content quality) are interpreted with nuance rather than essentialism. The significant correlations between maternal education, home literacy environment, and narrative quality add important sociocultural depth.

 The pedagogical implications—gender-sensitive rubrics, cross-gender writing exercises, and teacher training—are specific, feasible, and directly derived from the data. The article speaks clearly to both researchers and practitioners.

 No further revisions are necessary. The manuscript is clear, ethically sound, and makes a meaningful contribution to inclusive education literature.

**Recommendation:** **Accepted**

Is the work clearly and accurately presented and does it cite the current literature?

Yes

If applicable, is the statistical analysis and its interpretation appropriate?

I cannot comment. A qualified statistician is required.

Are all the source data underlying the results available to ensure full reproducibility?

Yes

Is the study design appropriate and is the work technically sound?

Yes

Are the conclusions drawn adequately supported by the results?

Yes

Are sufficient details of methods and analysis provided to allow replication by others?

Yes

Reviewer Expertise:

NA

I confirm that I have read this submission and believe that I have an appropriate level of expertise to confirm that it is of an acceptable scientific standard.

# Reviewer report: Reviewer response for version 2

**DOI:** 10.5256/f1000research.195218.r481049

**Published:** 2026-05-05

**Authors:** Ifigeneia Dosi

**Affiliations:** 1 Democritus University of Thrace, Komotini, Greece

The revised manuscript shows substantial improvement and addresses most of the major concerns raised in the initial review, particularly in clarifying research questions, strengthening methodological transparency, and incorporating SES variables and statistical analysis. The addition of coding details and inter-rater reliability enhances the study’s rigor. However, some concerns remain regarding moderate reliability, limited justification of the narrative task, and continued overinterpretation of gender differences despite non-significant statistical results. The authors may also consider engaging with additional recent research on children’s narrative writing and literacy development (refer 1,2, ) to further strengthen the theoretical grounding.

Is the work clearly and accurately presented and does it cite the current literature?

Partly

If applicable, is the statistical analysis and its interpretation appropriate?

Not applicable

Are all the source data underlying the results available to ensure full reproducibility?

No

Is the study design appropriate and is the work technically sound?

No

Are the conclusions drawn adequately supported by the results?

Partly

Are sufficient details of methods and analysis provided to allow replication by others?

No

Reviewer Expertise:

language acquisition; bilingualism, applied linguistics

I confirm that I have read this submission and believe that I have an appropriate level of expertise to confirm that it is of an acceptable scientific standard, however I have significant reservations, as outlined above.

## References

[1] : Beyond Monolingual Norms: Analyzing the Complexity of Greek-Turkish Bilingual Children’s Written Narratives. *New Perspectives on Languages* .2024; 10.56395/npl.v1i1.12 10.56395/npl.v1i1.12

[2] : Empowering Narrative Writing Competence in Elementary Education. 10.4018/979-8-3693-8931-7.ch011 211-230 10.4018/979-8-3693-8931-7.ch011

# Reviewer report: Reviewer response for version 1

**DOI:** 10.5256/f1000research.189228.r437062

**Published:** 2025-12-27

**Authors:** Ifigeneia Dosi

**Affiliations:** 1 Democritus University of Thrace, Komotini, Greece

This manuscript addresses an important topic—the relationship between gender and narrative writing in elementary school students—and positions itself within inclusive and critical literacy frameworks. While the topic is timely and potentially valuable, the paper suffers from substantial methodological, analytical, and interpretive weaknesses. These issues undermine the validity of the findings and lead to conclusions that are not adequately supported by the data. The concerns outlined below follow the structure of the manuscript and should be addressed to improve clarity, rigor, and interpretability.

**1. Research aims and research questions**

 The study formulates a broad research question concerning how gender norms and social values influence narrative writing styles and their implications for inclusive education. However:

- The research question is too broad relative to the scale and design of the study.
- No sub-questions are articulated, making it difficult to track how specific findings address distinct components of the question.
- Later sections of the paper do not explicitly return to the research question, nor do they systematically link findings to it.

A clearer articulation of research questions—and a more explicit mapping between questions and results—would substantially improve the coherence of the paper.

**2. Participants and background variables**

**2.1 Interview subsample**

 Only 10 out of 33 students were interviewed, selected purposively based on “distinctive gendered writing patterns.” However:

- The rationale for interviewing exactly 10 participants is not justified.
- There is no discussion of data saturation, representativeness, or alternative sampling strategies.
- Selecting interviewees based on pre-identified gendered patterns risks confirmatory bias, reinforcing the authors’ theoretical expectations.

**2.2 Socioeconomic status and literacy background**

 The manuscript provides no information about:

- Socioeconomic status (e.g., parental education, occupation)
- Home literacy environment
- Prior literacy instruction or writing experience

This is a serious limitation, as SES and literacy background are well-established predictors of narrative development and may confound the interpretation of gender-based differences.

**3. Narrative task selection**

 The study uses a personal narrative task (“Vacation Experience”), but the choice is not adequately justified.

- No rationale is provided for not using standardized narrative instruments (e.g., MAIN, ENNI), which would allow comparison with existing literature.
- Personal narratives are highly sensitive to cultural experience, SES, and emotional expressiveness, none of which are controlled for.
- The task inherently privileges reflective and emotional storytelling, which may bias results against action-oriented narratives.

As a result, it is unclear whether observed differences reflect narrative competence, task affordances, or assessment bias.

**4. Coding and measurement of narratives**

**4.1 Macro- and microstructure**

 The authors claim to analyze narrative structure at multiple levels, but this is done inconsistently and without clear operationalization.

- Macrostructure is not defined using established story grammar frameworks.
- Microstructure is reduced to surface features (sentence structure, spelling), without measures such as lexical diversity, syntactic complexity, or cohesion indices.
- The category “language” is insufficiently specified: it is unclear whether it refers to grammaticality, lexical richness, or syntactic organization.

**4.2 Coding procedures**

 The coding process lacks transparency:

- Coding categories are not clearly defined.
- No concrete examples of coded narratives are provided.
- There is no evidence of coding manuals or decision rules.

These omissions limit replicability and raise concerns about analytic validity.

**5. Assessor blinding and reliability**

 The manuscript does not report whether assessors were blind to participant gender.

- Given the handwritten narratives, gendered self-references, and the study’s explicit focus on gender, assessors likely knew participants’ gender.
- This is particularly problematic given the use of subjective scoring criteria (e.g., emotional depth, cohesion).
- No inter-rater reliability statistics (e.g., Cohen’s κ, ICC) are reported.

The absence of blinding and reliability metrics represents a major threat to internal validity.

**6. Results and interpretation**

 Although results are presented with numerical scores, the study does not conduct statistical testing, yet repeatedly refers to “differences” as if they were inferentially meaningful.

 More importantly:

- Descriptive findings are consistently interpreted through strong theoretical lenses (hegemonic masculinity, feminine scripts) without sufficient empirical grounding.
- Children’s brief interview statements are often over-interpreted using adult sociological frameworks.

This results in conceptual overreach, where interpretation exceeds what the data can reasonably support.

**7. Discussion structure and alignment with findings**

 The Discussion section is not well structured.

- It does not systematically follow the Results.
- Key claims are repeated across sections with limited analytical progression.
- The discussion blends interpretation, literature review, and normative recommendations without clear boundaries.

Critically, the discussion does not clearly demonstrate how the findings answer the stated research question, nor does it sufficiently acknowledge alternative explanations for the observed patterns.

 In its current form, the manuscript raises important questions but does not meet the standards of methodological rigor, analytic transparency, or interpretive restraint required for publication. Major concerns relate to participant characterization, task selection, coding procedures, assessor bias, and overgeneralization of findings.

Is the work clearly and accurately presented and does it cite the current literature?

Partly

If applicable, is the statistical analysis and its interpretation appropriate?

Not applicable

Are all the source data underlying the results available to ensure full reproducibility?

No

Is the study design appropriate and is the work technically sound?

No

Are the conclusions drawn adequately supported by the results?

Partly

Are sufficient details of methods and analysis provided to allow replication by others?

No

Reviewer Expertise:

language acquisition; bilingualism, applied linguistics

I confirm that I have read this submission and believe that I have an appropriate level of expertise to state that I do not consider it to be of an acceptable scientific standard, for reasons outlined above.

# Response

**Published:** 2026-03-12

**Authors:** Sri Wulan Anggraeni

**Affiliations:** Faculty of Education Sciences, Universitas Pendidikan Indonesia Fakultas Ilmu Pendidikan, Bandung, West Java, Indonesia

Research Objectives and Questions

 Specific questions have been added, and the results and discussion sections are now aligned with these questions to clarify the relationship between objectives, findings, and interpretations.

 Participants and Background Variables

 The purposive sampling of ten interview participants is now more justified, including considerations of data saturation and representativeness. Data on socioeconomic status (SES) and home literacy environment from the parent questionnaire have been included.

 Background SES and Literacy

 Additional SES information (parental education, occupation, and home literacy resources) has been added. Table 1 shows respondent characteristics by gender, and Table 7 presents the correlation between SES indicators and final scores.

 Narrative Task Selection

 The rationale for using the personal narrative task has been clarified as being developmentally appropriate and reflecting classroom practices consistent with the Indonesian curriculum. For narrative evaluation, this study adopted the narrative assessment framework developed by Burhan Nurgiyantoro (an Indonesian linguist and literary expert), which is widely recognized in educational research in Indonesia.

 Coding and Measurement

 The coding procedure has been refined with clearer categories, examples, and decision rules to increase transparency and replicability.

 Rater Deception and Reliability

 This manuscript now reports rater deception and inter-rater reliability statistics using Cohen's Kappa.

 Results and Discussion

 Statistical analyses (normality tests and independent samples t-tests) have been included, and the discussion section has been reorganized to align with the results and research questions.
